# Understanding the benefits and burdens associated with a malaria human infection study in Kenya: experiences of study volunteers and other stakeholders

**DOI:** 10.1186/s13063-021-05455-7

**Published:** 2021-07-26

**Authors:** Primus Che Chi, Esther Awuor Owino, Irene Jao, Fredrick Olewe, Bernhards Ogutu, Philip Bejon, Melissa Kapulu, Dorcas Kamuya, Vicki Marsh, CHMI-SIKA Study Team, Abdirahman I. Abdi, Yonas Abebe, Agnes Audi, Peter Billingsley, Peter C. Bull, Mainga Hamaluba, Zaydah de Laurent, Susanne H. Hodgson, Stephen Hoffman, Eric James, Gathoni Kamuyu, Silvia Kariuki, Nelson Kibinge, Rinter Kimathi, Sam Kinyanjui, Cheryl Kivisi, Nelly Koskei, Mallika Imwong, Brett Lowe, Johnstone Makale, Kevin Marsh, Khadija Said Mohammed, Moses Mosobo, Sean C. Murphy, Linda Murungi, Jennifer Musyoki, Michelle Muthui, Jedidah Mwacharo, Daniel Mwanga, Joyce Mwongeli, Francis Ndungu, Maureen Njue, Patricia Njuguna, George Nyangweso, Domitila Kimani, Joyce M. Ngoi, Janet Musembi, Omar Ngoto, Edward Otieno, Faith Osier, James Oloo, Donwilliams Omuoyo, John Ongecha, Martin O. Ongas, Michael Ooko, Jimmy Shangala, Betty Kim Lee Sim, Joel Tarning, James Tuju, Juliana Wambua, Thomas N. Williams, Markus Winterberg

**Affiliations:** 1 Centre for Geographic Medicine Research (Coast), Kenya Medical Research Institute-Wellcome Trust Research Programme, Kilifi, Kenya; 2 Centre for Clinical Research, Kenya Medical Research Institute, Kisumu, Kenya; 3 Center for Research in Therapeutic Sciences, Strathmore University, Nairobi, Kenya; 4 Centre for Tropical Medicine and Global Health, Nuffield Department of Medicine, University Oxford, Oxford, UK

**Keywords:** Human infection studies, Challenge studies, Controlled human infection studies, Burdens, Benefits, Developing countries, Ethics

## Abstract

**Background:**

Human infection studies (HIS) that involve deliberately infecting healthy volunteers with a pathogen raise important ethical issues, including the need to ensure that benefits and burdens are understood and appropriately accounted for. Building on earlier work, we embedded social science research within an ongoing malaria human infection study in coastal Kenya to understand the study benefits and burdens experienced by study stakeholders in this low-resource setting and assess the wider implications for future research planning and policy.

**Methods:**

Data were collected using qualitative research methods, including in-depth interviews (44), focus group discussions (10) and non-participation observation. Study participants were purposively selected (key informant or maximal diversity sampling), including volunteers in the human infection study, study staff, community representatives and local administrative authorities. Data were collected during and up to 18 months following study residency, from sites in Coastal and Western Kenya. Voice recordings of interviews and discussions were transcribed, translated, and analysed using framework analysis, combining data- and theory-driven perspectives.

**Findings:**

Physical, psychological, economic and social forms of benefits and burdens were experienced across study stages. Important benefits for volunteers included the study compensation, access to health checks, good residential living conditions, new learning opportunities, developing friendships and satisfaction at contributing towards a new malaria vaccine. Burdens primarily affected study volunteers, including experiences of discomfort and ill health; fear and anxiety around aspects of the trial process, particularly deliberate infection and the implications of prolonged residency; anxieties about early residency exit; and interpersonal conflict. These issues had important implications for volunteers’ families, study staff and the research institution’s reputation more widely.

**Conclusion:**

Developing ethically and scientifically strong HIS relies on grounded accounts of volunteers, study staff and the wider community, understood in the socioeconomic, political and cultural context where studies are implemented. Recognition of the diverse, and sometimes perverse, nature of potential benefits and burdens in a given context, and who this might implicate, is critical to this process. Prior and ongoing stakeholder engagement is core to developing these insights.

**Supplementary Information:**

The online version contains supplementary material available at 10.1186/s13063-021-05455-7.

## Background

### Human infection studies

Human infection studies (HIS), or human challenge studies, involve deliberately infecting healthy volunteers with a pathogen to understand disease pathogenesis and host immune responses, and to test the effectiveness of new vaccines and drug product [1–3]. Most HIS are conducted under controlled conditions in which a specific strain, dose and route of administering a pathogen under investigation are used; the pathogen is contained to minimise third party risks; and the extent of infection is limited based on understanding the pathogen and the availability of effective treatment [4]. In most cases, HIS volunteers are required to stay in an in-patient facility to enhance their safety through close clinical monitoring and to minimise third party risks, especially in cases where the challenge pathogen is contagious.

The primary rationale for HIS is based on a capacity to provide early indications of effectiveness for candidate vaccines or drug products, with potential to accelerate development processes. Additionally, since HIS tend to involve fewer participants in a controlled environment — in contrast to large-scale clinical trials — they can be conducted over shorter durations and support the testing of several candidate vaccines or drug products, translating to substantial cost-saving in vaccine and drug development initiatives. Of direct relevance to this paper, HIS are seen to have particular social value (including translational benefits) in settings where target diseases are endemic, since research populations are likely to have similar genetic and immunological profiles as a future target population. In these settings, prior exposure may be associated with less severe symptoms and the illness seen as an ‘everyday occurrence’ [5, 6]. Since HIS can help to address global burdens of vaccine-preventable diseases, their conduct in countries that carry the highest burden of these conditions is potentially important [3, 7, 8]. Although many HIS have been conducted in the high-income countries in the past decades, more recently they have been increasing number of HIS also being conducted in low- and middle-income country (LMIC) settings [9–11]. This recent increase in the number of HIS in LMICs may be associated with advances in research infrastructure, technical expertise and clinical facilities; more accommodating ethics and regulatory environment; and changing cultural norms [12].

### Malaria human infection studies

Malaria is a life-threatening disease presenting a global public health challenge and a leading cause of death in many low-resource countries [13]. Amongst malaria species, illness caused by infection with *Plasmodium falciparum* is responsible for the highest global burden of disease and deaths (94% and 95% respectively) and particularly impacts sub-Saharan Africa and children under 5 years [13]. Despite efforts to scale up preventive and treatment measures such as insecticide-treated bed nets, indoor residual spraying and use of antimalarial drugs, the global malaria burden remains problematic, particularly in the face of developing insecticide and drug resistance. The development of effective drugs and vaccines is an important global health strategy.

Malaria HIS (mHIS) involve deliberately infecting healthy volunteers with *falciparum* malaria through direct mosquito bites or injecting malaria sporozoites, towards understanding pathogenesis and host immune responses, or evaluating new malaria drug products and vaccine candidates. In endemic settings, mHIS volunteers are generally admitted to a residential facility for close clinical monitoring to enhance volunteers’ safety and avoid natural malaria infection.

### Ethical issues in human infection studies

A number of ethical issues have been raised across the literature [1], including the acceptability of deliberately infecting healthy volunteers with a pathogen [14–19]; the need to ensure social value (inform important issues for the host community) in a given context, given the harms and burdens that might be involved and the lack of direct therapeutic benefits to volunteers [20, 21]; and considerations around third party risks [4, 18, 22, 23]. Risks of undue inducement arise as prolonged periods of residency may attract substantial levels of monetary compensation [5, 24], such that volunteers may overlook potential risks and burdens, and studies attract the most-poor within a population [2, 21, 25]. Under-representation of the most-poor has also been raised as a fairness issue, linked to requirements for high literacy levels amongst volunteers given the complex nature of these studies. As a result, particular attention has been paid to fair and strong informed consent and engagement processes to ensure understanding of the social value, risks and burdens of participation [21].

In addition to risks for study participants, ethical issues may arise around reputational loss for researchers and institutions involved, particularly where unanticipated forms of harm arise [14, 15, 26, 27]. Where research institutions are linked to a public health facility, health service delivery may be negatively impacted by loss of trust.

### Malaria human infection studies in Kenya

**Fig. 1:**
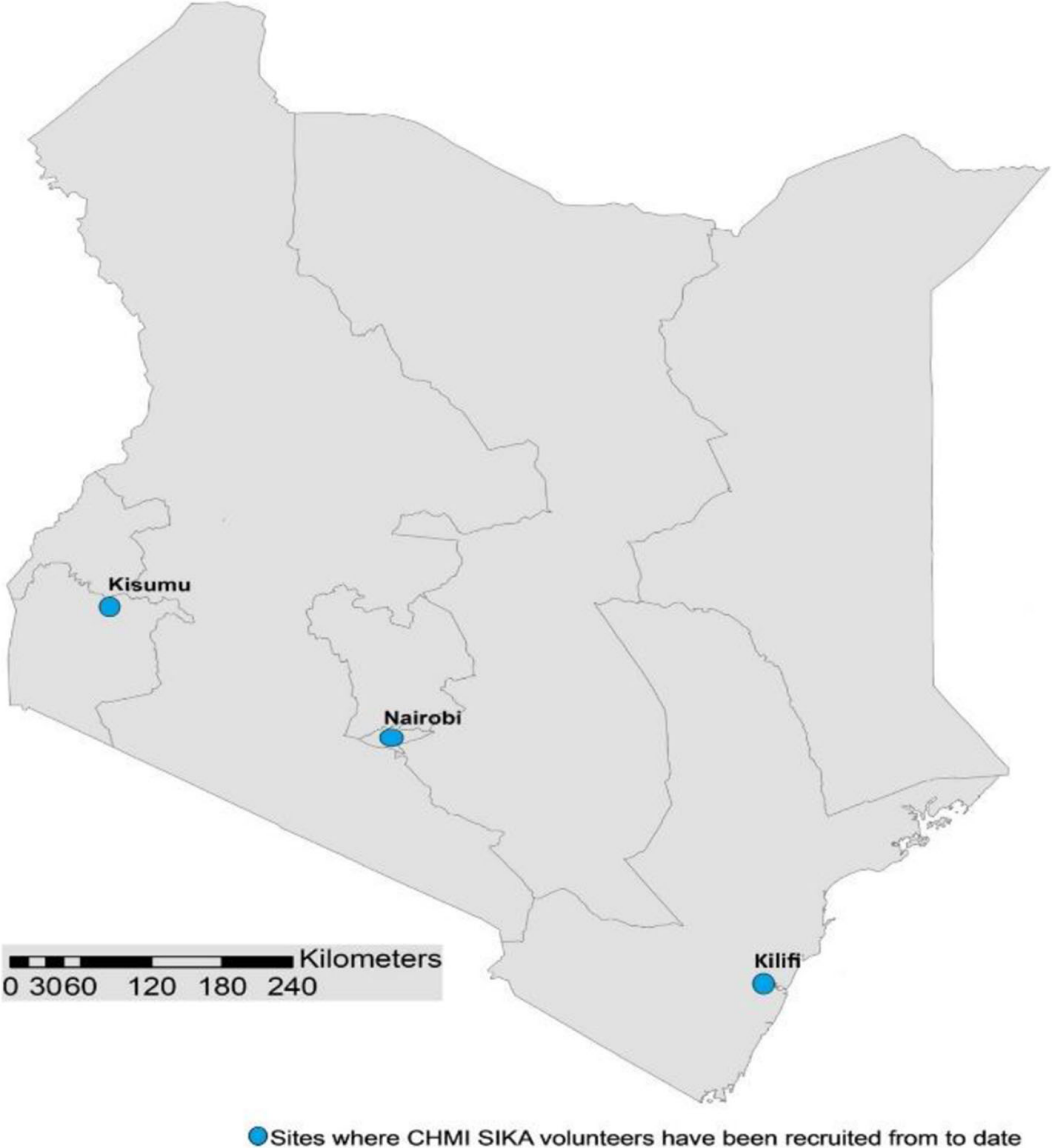
Geographical location of CHMI study sites in Kenya (Source: [14])

In Kenya, two mHIS have been conducted at the Kenya Medical Research Institute (KEMRI)-Wellcome Trust Research Programme (KWTRP) since 2013. The first was an open-label randomised trial of *Plasmodium falciparum* malaria in Nairobi, aiming to establish and assess the feasibility of this approach [3, 28]. The study involved intramuscular injection of *P. falciparum* sporozoites (Good Manufacturing Process certified) into 28 healthy Kenyan adults, with varying levels of prior malaria exposure. The second was an open-label, non-randomised trial conducted at KWTRP in Kilifi on the Kenyan coast, aiming to understand the relationship between host immunity and parasite growth [5]. This study involved exposing 161 healthy Kenyan adults, from different parts of the country and with varying levels of immunity, to *P. falciparum* through intravenous sporozoite injection. Two phases of the second mHIS involved healthy volunteers from Kilifi (with low and moderate levels of prior exposure to malaria) and from Kilifi and Ahero in Western Kenya (with high prior exposure to malaria) respectively. The second phase was conducted in collaboration with the KEMRI Centre for Clinical Research’s (CCR) Ahero Clinical Trials Unit (ACTU) in Kisumu^1^, who took responsibility for engagement and recruitment in Western Kenya. Figure 1 shows a map of Kenya, indicating the positions of these sites.

**Fig. 2:**
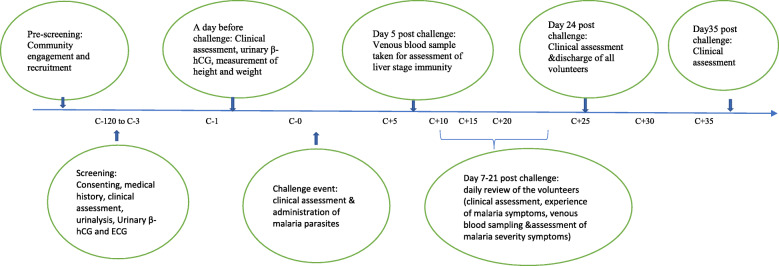
Kilifi malaria HIS procedures. Pre-screening, 3–4 months before screening 1st screening (Sep 2017); residency/in-patient stay, maximum 24 days after challenge; 2nd screening, 3–4 months after 1st screening (Jan/Feb 2018); review clinic, 35 days post challenge; challenge event, 5 months after 1st screening (Feb/March 2018)

The main procedures for the second phase of the mHIS study conducted in Kilifi are illustrated in Fig. 2. The study start date was delayed by the announcement of presidential elections in Kenya that took place in August 2017. While community and stakeholder engagement and volunteer screening were initiated in Kilifi and Ahero in late 2017, the main study activities were postponed to January 2018, when volunteers were re-screened [5]. Eligible volunteers recruited into the study took up residence for a period of up to 24 days in the guest house of a local university immediately following their ‘challenge’ with a malaria sporozoite injection at KWTRP clinical facilities.

During residency, a dedicated KWTRP clinical team monitored volunteers closely, particularly for clinical malaria, being present throughout the day and available at night. Following the challenge event, 62.6% volunteers subsequently developed malaria (that is, had parasite positive tests with or without clinical symptoms), with varying severity of symptoms and after different periods of time. All volunteers who developed malaria post challenge were treated and discharged once symptoms had resolved and malaria tests were negative. Those who reached day 21 post challenge (C+21 in Fig. 2) without experiencing malaria were also given anti-malaria treatment and discharged on day 24 (C+24). At the end of residency, each volunteer was given a cash compensation for their time (approximately 20 USD per day) related to the total number of overnight stays [21]. All volunteers were asked to attend a study clinic for review on day 35 post challenge, with volunteers reviewed from the site where they were recruited from.

### Embedded ethics and social science work in HIS studies in Kenya

The social science study described in this paper was embedded within the Kilifi mHIS, conducted in phases to follow the main study activities, as a form of empirical ethics research [29]. For clarity, in this paper, we refer to HIS participants as a whole as ‘volunteers’ and those who agreed to join our embedded social science study as ‘participants’. Two rounds of social science data collection (T1 and T2) occurred during the first HIS phase involving volunteers, during the residency period (T1) and within weeks of leaving residency (T2). A further round of data collection (T3) was held in 2019, to include volunteers. Some findings from data collection at T1 and T2 have been published elsewhere, addressing participants’ experiences of and understanding and motivation for participation [14].

In this paper, we explore participants and other research stakeholders’ experiences of benefits and burdens in relation to the mHIS in Kilifi and consider their implications, including for policy. Our research is underpinned by recognition that a grounded account of these experiences is critical to identifying the nature and importance of benefits and burdens, including their nature as physical, psychological, social, familial and/or economic issues [26, 30, 31]. The paper draws on findings from all three rounds of social science data collection (T1, T2 and T3). We aimed to characterise benefits and burdens from HIS volunteers’ and other study stakeholders’ perspectives, and consider ways in which benefits could be maximised and burdens minimised; a fundamental ethical requirement in health research [16–18, 32, 33]. Illustratively, the Declaration of Helsinki [16] calls on researchers to continuously monitor, assess and document the risks of study participation. *In a separate paper, we will consider the implications of these findings for risks of undue influence in relation to volunteers’ decisions to join the mHIS study*.

## Methods

### Study context/site

The mHIS challenge event, residency and main laboratory work were undertaken at KWTRP in Kilifi, in collaboration with the KEMRI Centre for Clinical Research (CCR). KWTRP is an international collaborative long-standing and multidisciplinary research programme hosted by the Kenya Medical Research Institute, a semi-autonomous government organisation^2^ [5, 14, 21]. The programme has a long-standing platform for community engagement involving locally elected community representatives, leaders and other community stakeholders across the county [34, 35]. Kilifi County is located in the coastal region of Kenya whereas Ahero, Kisumu County, lies in the Western part of the country along the shores of Lake Victoria as shown in Fig. 1. The two counties are 843.9 km apart, a distance that would involve a 14 hours’ drive by road. Communities within Kilifi are mainly from the Mijikenda ethnic group, with a majority living in rural areas characterised by high levels of poverty and illiteracy [34]. The county’s economy is fuelled by subsistence farming, fishing, tourism and trade in the urban centres. Residents in Kisumu County are predominantly from Luo ethnic groups; major economic activities include subsistence and commercial farming, fishing, trade and light industries.

### Study participants

Table 1 summarises information on study participants, the timing and nature of data collection, the form of purposive sampling adopted and data collection methods (including research team members’ roles in data collection and analysis).

**Table 1:** Summary of data collection activities

Timing and site	2018: February–April (T1) Kilifi	2018: May (T2) Kilifi	2019: May–July (Kilifi), September (Ahero) (T3)
**Study participants**	• Study volunteers: Kilifi (*n* = 17) and Ahero (*n* = 15) • Study investigators/ clinicians (*n* = 3) • Kilifi study fieldworkers (*n* = 14) • Kilifi KCRs (*n* = 20)	• Former study volunteers from Kilifi (*n* = 5)	• Former study volunteers in Kilifi (*n* = 8) and Ahero (*n* = 10) • Study investigators/ clinicians (*n* = 5) • CLG Kilifi (*n* = 3) and Ahero CE staff (*n* = 1), • Kilifi study fieldworkers (*n* = 8) and Ahero CHVs (*n* = 4) • Community leaders in Kilifi (*n* = 3) and Ahero (*n* = 2)
**Purposive sampling criteria**	All staff and study volunteers at in-patient stay with the exception of those who were experiencing malaria symptoms	Diversity in social and research experiences during residency	Diversity in gender, location, time of diagnosis and duration of in-patient stay
**Data collection methods (data collection team)**	IDIs (*n* = 3) with study staff; FGDs with fieldworkers (*n* = 3), volunteers (*n* = 3) and KCRs (*n* = 3) (IJ/DK)	Follow-up IDIs (*n* = 5) (2 involved in residency FGDs) (IJ)	IDIs (*n* = 28) and pairs interviews (*n* = 8; with 16 persons) (EO/PCC)
**No. of study participants**	66	5 (2 participated in FGDs at T1)	44 (16 participated in FGDs at T1)

*CLG* Community Liaison Group, *CE* community engagement, *CHVs* community health volunteers, *KCRs* KEMRI community representatives, *IDIs* individual interviews, *FGDs* focus group discussions

Overlaps occurred across data collection activities as follows: two Kilifi volunteers in follow-up interviews at T2 also participated in FGDs at T1, three Kilifi volunteers and all Ahero volunteers in T3 participated in FGDs at T1, and three Kilifi fieldworkers in T3 participated in FGDs at T1

Data from 97 participants are drawn on in this paper, including HIS volunteers (*n* = 37); study staff, including investigators, clinicians, fieldworkers/community health volunteers and community engagement staff (*n* = 35) and community representatives, including KEMRI community representatives (KCRs) and leaders (*n* = 25). Data were collected at T1 (April 2018) and T2 (May 2018) in Kilifi and at T3 (May to September 2019) in Kilifi and Ahero. Most of the data presented^3^ were collected during the third period (T3).

Table 2 summarises sociodemographic information for HIS volunteers involved in this study, while Table 3 describes gender, roles and residential areas for research and community stakeholders. The mHIS only recruited volunteers aged 18 to 45 years [5]. In this qualitative study, participants were sampled purposively, aiming to maximise diversity in sociodemographic features and life experiences of volunteers, as shown in Table 1.

**Table 2:** Demographic characteristics of study participants

Location	Kilifi		Ahero		Total (%)^**#**^
Characteristics	Male	Female	Male	Female	
**Age (years)**^α^					
19–29	3	5	4	3	15 (40.5)
30–40	6	3	3	3	15 (40.5)
41–51	2	3	1	1	7 (18.9)
Education level*					
None	0	2	0	0	2 (5.4)
Primary	5	5	1	1	12 (32.4)
Secondary	3	1	4	4	12 (32.4)
Tertiary	0	1	3	2	6 (16.2)
Unavailable	3	2	0	0	5 (13.5)
**Occupation**					
None	3	4	0	1	8 (21.6)
Student	0	0	3	0	3 (8.1)
Subsistence farming	1	5	0	0	6 (16.2)
Self-employed/business	5	1	5	5	16 (43.2)
Employed	2	1	0	1	4 (10.8)
**Total**	11	11	8	7	37 (100)

^α^The mHIS only recruited healthy volunteers aged 18–45 years for safety reasons

*Information on occupation for 5 volunteers is missing

^#^% are rounded to the nearest 10th, hence % may not add up to 100

**Table 3:** Distribution of other stakeholders across the data collection periods by location and sex

Category of stakeholder	T1		T3			
	Kilifi only		Kilifi		Ahero	
	Male	Female	Male	Female	Male	Female
Investigators/clinicians	1	2	0	2	3	0
FWs/CHVs	10	4	4	4	1	3
CLG/CE staff			2	1	0	1
Chiefs			2	1	2	0
KCRs	12	8	0	0	0	0
Total	23	14	8	8	6	4
	37		26 (3 FWs participated in FGDs at T1)			

*FWs* fieldworkers, *CLG* Community Liaison Group, *CE* community engagement, *CHVs* community health volunteers, *KCRs* KEMRI community representatives

### Data collection

#### Data collection during T1 and T2

During residency (T1) and immediate follow-up (T2), data were mainly collected through observations, in-depth interviews (IDIs) and focus group discussions (FGDs), as shown in Table 1. Non-participant observations were conducted during early community engagement activities, intermittently throughout residency and during the final follow-up visits for the mHIS (C+35). IDIs with staff and volunteers FGDs were held in a private room within the HIS residential facility. All FGDs with the fieldworkers and Kilifi KCR were conducted within the community, at local health dispensaries. IDIs with Kilifi volunteers at T2 took place at local community health dispensaries, with the exception of one at the participant’s home. Community representatives in Kilifi were drawn from an existing platform, the KEMRI community representatives (KCRs) that have been described elsewhere [34] — for engagement, to involve residents from the geographic location from which volunteers had been recruited.

#### Data collection during T3

During T3, data were collected in Kilifi and Ahero. As shown in Table 1, data collection methods included individual in-depth interviews (study staff and HIS volunteers), and pairs interviews [36] (Kilifi fieldworkers, Kilifi CLG staff, Ahero CHVs, administrative leaders).

All data from Ahero-based participants at T3 were collected at the Ahero Clinical Trials Unit (ACTU) in Western Kenya, the local study site. In Kilifi, former HIS volunteers were interviewed at their nearest health facilities, while study staff and community leaders were interviewed at KWTRP offices. All interviews (individual and pairs) lasted between 30 to 90 min, and FGDs between 80 and 180 min. Data were generated through the use of topic guides (see Additional file 1) designed to encourage a narrative account of volunteers’ experiences of the HIS over time, with a focus on aspects of the study that were appreciated or seen as difficult or challenging. In each time period, data collection tools were piloted, and — as is typical for qualitative research — revisions made during data collection to allow a focus on emerging issues.

### Data analysis

All data from individual and pairs interviews and FGDs were audio recorded, transcribed verbatim and translated to English, where indicated, by experienced transcribers and translators within the KWTRP social science group, and checked by IJ or EO for accuracy. Note takers present during interviews and FGDs contributed to the early stages of analysis.

Data analysis was guided by the framework analysis approach [37] drawing on both deductive (a priori, from the literature) and inductive (led by the data) processes. Using this approach, a small sample of rich transcripts were identified and used to develop an initial coding framework in T1/2 and in T3, at separate times. Initial coding frameworks in all time periods were further developed through application to all the data, following an iterative process supported by study team discussions, leading to the final framework applied. Interpretation of the data involved the identification of themes across coded data and subsequent tabulation of themes by participants or groups to identify patterns across the data, as an analysis chart. The final data set for this paper draws on T1/2 and T3, through the merging of data from the earlier period into the charts developed during the second period (T3). IJ and EO/PCC took a lead on coding processes in T1/2 and T3, respectively, and DK and VM were involved in analysis and interpretation in T1/2 and T3, respectively. Data were managed using QSR NVivo software.

## Findings

Given the focus in this paper on giving an in-depth account of the nature of benefits and burdens experienced by HIS volunteers and other study stakeholders, we present the findings under broad themes related to these two main topics, which are summarised in Table 4.

**Table 4:** A summary of reported benefits and burdens for volunteers and other stakeholders in the malaria HIS

Area	HIS volunteers: benefits/aspects of participation valued	HIS volunteers: burdens/aspects of participation seen as problematic	Spread of individual benefits/burdens to community, research staff and institution
Preparation and travel to study site for residency	None described	Tiredness and anxiety associated with long distance travelled, perceptions of safety (political situation) and of inadequate time to prepare for travel to take up residence	Loss of trust and reputational damage to institution, particularly in relation to political tensions
Access to a health check	*Physical and psychological benefits* • Health check valued particularly if found to be ‘normal’ • Potential benefit from detection of underlying health problems and referral for care	The possibility of ‘failing’ a health check caused anxiety related to loss of opportunity to participate (and benefit from compensation) and risks of stigma, where latter widely seen as linked to HIV status	Rumours/stigma related to perceptions that people who are excluded from the malaria HIS on the health test are HIV positive
The malaria challenge and follow-up ‘clinical’ processes	None described	*Physical and psychological burdens:* • Pain and discomfort caused by intravenous injection of malaria sporozoites and later blood sampling • Anxiety (sometimes severe) about risks involved in short and longer term, linked to issue of trust in research and researchers (generally but not always temporary)	Issues of trust around researchers’ intentions and short- and long-term effects of research procedures can spread to community as rumours (positive or negative); negative attitudes towards research staff (including field workers who are based in the community); potential institutional reputational damage; and impact on other studies
Compensation	*Social and economic benefits* Compensation highly valued and in practice generally used in ways that promoted individual or family wellbeing in the long term, often in modest ways.	*Social and psychological burdens* • Anxiety (sometimes severe) about minimisation of compensation in the event of early study exit • Family conflicts over decision to join study (to gain compensation) • Family conflicts over the way compensation should be used, with gendered dimension	Conflicts in families are likely to generate issues within the wider community, potentially leading to community conflict, community-researcher trust issues and risks to institutional reputation and other studies.
Residential experience	Social and psychological benefits • Facilities enjoyed as ‘paid for vacation’ • New friendships valued, sometimes long lasting • Other opportunities for learning during residency valued e.g. tour of labs	Social and psychological burdens: • Families missed and worried about, particularly in relation to their economic and health status while volunteer away, particularly if had role as main ‘breadwinner’ • Offence to other residents and family/community conflict caused through development of relationships seen as ‘inappropriate’ • Boredom and frustration experienced	
Being forced to use contraceptives	None reported	Psychological burdens for women volunteers — disliked as ‘not normal’ practice, fears about safety and concerns about being forced to use	
Opportunity to contribute to new vaccine development	A valued opportunity given the nature of malaria as a well-recognised and serious illness in local communities	As above, anxiety (sometimes severe) about risks involved in short and longer term, linked to issue of trust in research and researchers (generally but not always temporary)	

### Perceived ‘benefits’

A range of features or experiences of participation in the malaria HIS were valued, including social, economic, psychological and physical benefits. The cash payment provided as compensation for time and the residential conditions were most commonly discussed as benefits in ways that suggested their overall importance to volunteers.

#### Compensation for study participation

Across all three data collection periods, nearly all study participants talked about compensation as the main reason they decided to join this study, and those who described other more important reasons for joining had also been highly motivated to join by the lump sum provided at the end of residency. Reflecting its value, many had planned how they would use this lump sum beforehand. As described earlier, levels of compensation were tied to the number of days spent in residency during the mHIS, with early exits prompted by episodes of clinical malaria requiring treatment. Volunteers, who did not develop malaria in response to the ‘challenge’ event or developed this later in the course of residency, generally received higher levels of compensation than those who exited the study earlier.

While a few seemed to feel that the cash payment was not particularly high, and some felt it should have been higher, the particular value attached to the cash payment was the certainty of its disbursement, given that many volunteers relied on uncertain or irregular livelihoods or were students (Table 2). Some volunteers described study participation as a form of employment or economic activity:

…I have come to realize that many of them [volunteers] join…having in mind that in one way or the other, it’s an economic activity, because there is the reimbursement which you get after the research. (Kilifi volunteer, male, IDI5, T3)

Across the data collection periods, several aspects of participants’ accounts reflected this high appreciation of the compensation provided, from their own and their families’ perspectives, including that (i) almost all participants described a sustained willingness (up to T3) to consider joining a similar challenge study in the future and (ii) some had either drawn on the compensation payments to convince reluctant family members to support their decision to join or had themselves been similarly convinced to join by family members:

For me it was not a problem because my husband knows that I am a person who is always looking [for money]. He knows that wherever I go, whatever small thing I get, it will help the children in school. He told me ‘Aah that is a good chance and if you lose it you won’t get another one. So if you feel you are healthy you can go’. (Kilifi volunteer, female, FGD6, T1)

#### Comprehensive health check

As part of the recruitment process for the HIS, all potential volunteers had agreed to a range of physical and laboratory tests in keeping with the overall inclusion and exclusion criteria of the study, summarised in Table 5.

**Table 5:** Screening tests

• Clinical observations: pulse, blood pressure, respiratory rate and temperature • Blood tests: ◦ Haematology: full blood count, screen for sickle cell trait and thalassemia ◦ Biochemistry: sodium, potassium, urea, creatinine, albumin, alanine aminotransferase (ALT) and bilirubin ◦ Diagnostic serology: HIV antibodies ◦ Immunological assays of prior exposure to malaria/assessment of immunity to malaria ◦ Diagnostic malaria tests (PCR and microscopy) • Urinalysis: for protein, glucose or blood cells • Urinary beta-Human Chorionic Gonadotrophin (β-hCG) for all females • Electrocardiogram (ECG)	

During all data collection periods, participants valued the health check during screening, including some who saw this as more important than the cash compensation. While the nature of the tests seemed not always to be fully understood, positive attitudes to testing were based on perceptions of thoroughness, high quality and accessibility, in contrast to tests outside the study.

…I also liked it because you know these tests it’s hard for someone to go to a hospital to get tested…so to some point I count myself lucky because I know it will be costly to me if I decide to do it on my own. But at least if somebody somewhere did it for me and at least I was confirmed ok, I am happy about it (Ahero volunteer, female, IDI17, T3)

As for the compensation provided, some volunteers drew on the health check to convince family members of the value of joining the study. Study fieldworkers felt that screening was generally seen as a positive experience, also pointing out that people screened out because of health conditions were often still positive, based on witnessing good progress amongst others subsequently referred for treatment. At the same time, the health check generated issues for volunteers, described in the later ‘Challenges (burdens) experienced in relation to malaria HIS participation’ section.

#### Good residential living conditions

The HIS volunteers mainly described residential conditions as a valued aspect of participation, across all time periods, and some were particularly appreciative. The residential experience was described as a form of high-quality paid-for vacation, with no work to do and time to rest. Specific aspects valued were the cleanliness of the environment, access to amenities like toiletries, ‘good’ meals being served regularly and on time and access to games for entertainment. In this way, while the main concern around staying in the study for as long as possible was related to maximising the compensation for time that would generate, the residential facilities themselves also seemed to provide an incentive for some to ‘stay in the study’.

…we were feeding well, sleeping well, the environment itself was ok. The food, hei! ...we used to eat so well and any kinds of food. You queue and serve whatever you feel like eating, the food was in plenty. Then the environment at XXXXX (name of place of residency), there were so many trees… there were some benches where you could go relax and chat with your friend, it was so nice. (Ahero volunteer, female, IDI13, T3)

KEMRI Community Representatives and HIS field workers had a similar impression of how these facilities were valued, including very positive reports given by volunteers on returning home, around the accommodation, food and games for entertainment. KEMRI fieldworkers remembered positive stories from volunteers, and their expressed willingness to join another similar study in the future, including a willingness to stay longer in the residence, if needed.

#### New learning opportunities

Several volunteers valued experiences of new learning during their in-patient stay, including learning about KEMRI and clinical research more broadly. A particularly appreciated activity during residency was a tour of the KEMRI laboratories, which also seemed to address concerns related to community-based rumours about the reasons for blood collection at the research organisation [34]. Volunteers were shown the large-scale freezers where samples are stored and laboratory benches where tests are run, giving insights into real life research and researchers, in contrast to media representations. Volunteers valued learning about malaria control approaches in the home as well as the malaria vaccine to which the HIS aimed to make a contribution. Some Kilifi-based volunteers appreciated learning about a science attachment programme at KWTRP as relevant to their own families^4^:

… for example when a student …has excelled in…sciences and chemistry… they get an opportunity with KEMRI people…I thought that it’s my responsibility…as a parent…to encourage my child to work hard in school so that when they get the qualifications needed to do research, then it will be luck to me (Kilifi volunteer, male, IDI5, T3)

#### Developing new friendships

A feature of residency that strongly coloured volunteers’ experiences was the opportunity provided to meet new people, including from different parts of the country. This was largely seen as an enjoyable aspect of participation, and in some cases these relationships developed into good friendships and even romantic relationships. At least one such relationships seemed to involve a serious commitment that the couple hoped could lead to marriage; the young man in this case intended to talk to his father on return home to take this plan forwards.

But my father knew I have come for a study, but not to get a girl to go back with! Maybe if I go back, I can tell him ‘Well, things happened there…what do you say?’… I am now of age and can be able to start my life (Ahero volunteer, male, FGD5, T1)

Volunteers drew strength and encouragement from friendships made during residency, especially during difficult periods. Activities enjoyed included ball games and faith-based groups, which kept spirits high and countered boredom. While we later describe challenges around interpersonal relations during residency, 12–18 months later, many former volunteers were still in touch with friends made at that time from other parts of the country.

#### Personal satisfaction in contributing to a new malaria vaccine

Volunteers described pride in contributing towards developing a malaria vaccine, an important public health need in their communities, as one of the most fulfilling aspects of participation and acting as an encouragement to stay in the study. At least one volunteer recognised complexities in the move from research to policy:

…if probably this vaccine is developed and it’s out, I would only maybe suggest or recommend… let it be at an affordable rate. (Ahero volunteer, female, IDI14, T3)

### Challenges (burdens) experienced in relation to malaria HIS participation

Challenges around involvement in the mHIS were described by a range of stakeholders, primarily for HIS volunteers themselves, but also for their families and wider communities. Additionally, study staff experienced important challenges through their work, and taken together, we note the emergence of risks for the research institution itself.

#### Challenges for study participants and their families

Across this section, challenges experienced by volunteers are discussed in approximate chronological order across study-related activities, noting their nature as physical, economic, social and psychological challenges and assessing their perceived importance where possible. Some challenges reflected the ‘flip side’ of a benefit; that is, quite severe concerns and anxieties were often related to the risk of losing anticipated benefits, and some benefits also had perverse implications, as described across the following sections.

##### Anxieties around health screening

While mHIS volunteers had met the eligibility criteria during health screening, this group shared fears and worries they had experienced around screening processes, including the implications of being found to have a previously unknown health condition.

…so I was worried, and I thought ‘will I really succeed?’ because they were testing blood, all the tests, and you know nowadays there are a lot of diseases, so I was worried (Ahero volunteer, female, IDI6, T3)

The period of worrying about the findings of health screening as prolonged by the staggered process (see Fig. 2), with a second screening immediately before the start of the malaria HIS. The main concern was a new diagnosis of HIV infection, including the direct implications for health and fears of stigmatisation by others in future. Although the research team made clear that people might be ‘screened out’ for many different reasons (not just HIV, as well as abnormal laboratory results) [5] — to counter risks of community stigmatisation around HIV — this risk still appeared real to many volunteers. Additionally, those diagnosed as HIV antibody-positive were referred to an appropriate health centre for further counselling and treatment. Some choose not to disclose their involvement in the study outside their immediate household until their enrolment had been confirmed. In relation to the health check, an additional concern was a lack of gender sensitivity, commented on by a woman who was uncomfortable being examined by a male clinician.

##### Preparing to take up residency at the in-patient study facility

**Stress in meeting family needs before leaving**

As study participants prepared to take up residency at the in-patient facility, many described the stress and practical difficulties involved in ensuring their families’ daily needs could be met over the residency period, especially those who were the main ‘breadwinners’ for their families. These needs included money for food, school fees and medical costs, and ways of maintaining normal income-generating activities, such as small-scale businesses. Many participants, particularly from Ahero, felt more time should have been given for this preparation. Others were challenged to secure the needs of their families for such a long period of residency ahead of time, irrespective of the time given to prepare. Worrying about families at home was a major burden for volunteers throughout residency.

**Insecurity, long travels and reception upon arrival**

Study participants from Ahero met challenges in travelling to Kilifi for re-screening and residency, given the distance involved and post-presidential election turmoil in this opposition stronghold, when some roads were unofficially blocked by protestors and an overall feeling of insecurity prevailed.

The journey was difficult. That day there were gunshots here at Ahero ...So even leaving the house, the journey was difficult, but we just went until we reached. Even the bus that we had boarded, some of the windows were broken. So, we were worried because of the chaos. (Ahero volunteer, female, IDI08, T3)

Before study activities were temporarily halted, security concerns were heightened by the juxta positioning of study recruitment and the presidential elections since both required access to potential volunteers’ identity cards and travel allowances were given to Ahero volunteers, leading to rumours that the study was recruiting young people for a politically related activity in Kilifi. Study staff in Ahero were also concerned about being targeted by angry protesters:

… there was a certain age [group] that we wanted…mostly youths so they [community members] could say they are taking our youths to Kilifi for maybe elections and … they thought that we are giving this one a thousand [travel costs] because we are convincing them to go Kilifi …to get numbers… we became worried … they could come and attack us (Field officer Ahero, female, IDI1, T3)

Given issues with travel, some Ahero participants were annoyed not to be given more time to recover between arrival in Kilifi and starting the re-screening process, an issue still talked about 18 months after the study. During re-screening on arrival in Kilifi, a few individuals were found to have been exposed to malaria so were excluded from the study and had to return home. While this late form of ineligibility was unplanned, being generated by the delay caused by political events, the anxiety, inconvenience and disappointment caused to a few was considerable.

##### Mandatory use of an effective contraceptive

Several women participants, especially from Ahero, described shock and concern about the enforcement of a study requirement to use an effective contraceptive to prevent pregnancy during the study, an issue that remained live 18 months later. According to the study protocol, an effective contraception is defined as a contraceptive method with failure rate of less than 1% per year when used consistently and correctly, in accordance with the product label [5]. While this requirement had been part of study information-giving processes, many had apparently not taken this seriously. Lack of previous use and the experience of unwanted side effects made this a difficult action. Volunteers’ reactions included regret at having joined the study, feeling forced to use a contraceptive against their will, and later non-adherence to this requirement:

…it slipped me on the first day of explanation…I was even trying to tell that lady ‘Why don’t we just write “condom”? Just write anything.’ And then she said ‘No, they don’t agree with the condom, you have to have the real, you see, family planning.’…when you have no choice, is that a motivation really? (Ahero volunteers, female, FGD4, T1)

…since birth I hadn’t used that thing (contraceptive), so I was being forced to use it and I also didn’t know why we were being forced to use it …so I said that I would get sick here and I won’t survive, if I take this thing twice I will hurt myself, so I took it once and stopped using it (Ahero volunteer, female, IDI18 T3).

##### The challenge event, subsequent study procedures and long-term health issues

**The challenge event: physical and psychological burdens**

As described in earlier publications, the challenge event was accompanied by important physical and psychological burdens, including physical discomfort involved in the intravenous injection of malaria sporozoites, the subsequent frequent venous and capillary blood sampling over time and, for some, the experience of developing a malaria infection [14, 21]. These burdens were increased by (generally short-lived) doubts about the truthfulness, motivation and professional standards of researchers, reflecting trust issues around this unfamiliar form of research [14]. A recurring example was sometimes quite marked worry about the nature of the challenge injection, including that it might contain a more ‘deadly’ pathogen like HIV or be contaminated, particularly as the injection was drawn up in a side room. Some volunteers expressed worries about contamination of the syringe. Other concerns related to long-term consequences, including that the parasites, would not be completely cleared by antimalarial treatment at the end of the study, reinforced by a study requirement for a post-study follow-up visit at day 35.

… suppose we get treated, but then there is some kind of resistance?... now I’m just wondering, suppose this resistance is there after day 35 who will take care of- of me? That is my main concern. (Ahero volunteer, male, FGD5, T1)

Other volunteers continued to worry about experiences of ill health long after participation, described a year later:

At times I feel like, especially my arms… they don’t want to be subjected to any strenuous work…they are fatigued…at times they are numb…[and] I can’t do anything… I’m just thinking could it be that those things that we were injected is what is affecting us this way? At times I just keep thinking, I’m just scared… (Ahero volunteer, female, IDI5, T3)

##### Family relations: missing and being unable to support families during residency

Being away from their families weighed heavily on many volunteers, especially women who had left young children at home. Worries included that children were suffering from neglect, lack of parental support and poor health, linked to difficulties experienced in finding a fully trusted caregiver for this period. One volunteer left the residency (and study) early for this reason, within the last 3 days of antimalarial treatment. In the study protocol, compensation payments for time in residency were to be given to volunteers on leaving the facility, as it was not possible to predict the duration of stay and a lumpsum payment was thought to be preferred by volunteers. But this lack of ‘cash in hand’ over the period compounded emotional experiences of missing family and familiar surroundings. Even where a trusted caregiver was in place, volunteers worried about providing basic necessities such as food and school fees at home, made worse by difficulties in maintaining mobile phone communication given the costs involved. Volunteers also felt the lack of cash in relation to their own ongoing needs that were not covered by the study, such visiting a barber. A few participants asked contacts at home to send money. Many HIS volunteers recommended that earlier disbursements of compensation payments should be made, rather than providing a lumpsum at the end.

I am the overall breadwinner and my family depends on me. So while I am away, I’m not happy to be eating well while I don’t know whether they got something or not, yeah. … and I cannot say that… I left something enough for them (Ahero volunteer, male, FGD5, T1)

Family relations had in some cases been challenged by volunteers’ decisions to join the study and withdraw from other responsibilities, which added to worries for some:

What I was talking about earlier about that hotel, it belongs to my sister. So, she said you just go, but if you go, we’ll never be in good terms again…because… I am the one who runs that hotel … So, from that date [of leaving home] up to today, she has never called me again. Even when I call her, she doesn’t receive my calls. (Ahero volunteer, male, FGD5, T1)

##### Worry about and actual impacts of residency on livelihoods and commitments

During residency, some volunteers expressed anxiety about the security of their prior livelihoods. Amongst these were individuals who had not informed their employers about joining the study and self-employed people with small businesses, who worried whether they would have businesses to return to, for example, whether regular clients would have found alternative suppliers:

One is my business. I have a business… I have chairs and tents. It is seriously affected because I’m not there. Secondly is … church issues…and I’m the chairperson. So… they seriously need me even now…it has really affected the organization. So that again I think it has affected me in a way because there was a duty that I was to perform. (Ahero volunteer, male, FGD5, T1)

Others were concerned about the impact on farming activities at that time, in relation to the year’s eventual harvest. While some were worried about their businesses and other activities at home, others were already receiving ‘bad’ news from home about mismanaged businesses while in residence.

Overall, in the long term, volunteers’ reports on livelihoods suggest that many participants found ways of using the compensation payments to enhance their lives in modest ways, including paying off debts, as we will describe in more detail in a future publication. Others did not appear to have benefitted and some described being ‘worse off’ in ways that reflected their concerns during residency. For example, at least one participant later lost their job because of the long absence while participating in the study.

##### Challenges during residency including interpersonal conflicts

While residency conditions were generally highly appreciated, a number of challenges were also associated with this aspect of participation. A common but short-term issue was the experience of boredom associated with restricted movement during this period, set up to promote compliance with scheduled study procedures and minimise the risks of volunteer’s developing new malaria infections. Many study participants described this confinement as very burdensome, especially young men, who made frequent requests for more freedom:

And even staying for those 21 days in that place, it’s not easy, it is hard….if we could go out for a walk a bit and come back, that would be better. But there is no leaving completely. It becomes so difficult that even the legs can get swollen. (Kilifi volunteer, male, FGD7, T1)

Underlining the extent to which some volunteers valued residency conditions, volunteers described worries about being forced to leave early (for volunteers who developed malaria soon after the deliberate infection) and more subtle concerns that the ‘comfortable’ residential living conditions would make settling back to demanding home routines difficult, highlighting the often physically arduous nature of ‘normal’ life:

I don’t know whether I’ll be having the strength which I’ve been having because with me I used to cook. So I don’t know if I’ll get that strength again [softly with voice trailing, sounding upset]. (Ahero volunteer, female, FGD4, T1).

The most important but relatively uncommon challenge related to residency was the emergence of interpersonal conflict amongst volunteers, as social relations within the facility were not always smooth. Several complaints were made by participants who were offended or inconvenienced by the apparent development of intimate relations between study participants. We described one such relationship in the section on benefits, in which the individuals involved made a serious commitment to each other. There were reportedly other instances that caused offence to participants, in which behaviours were judged immoral.

In the residency, sleeping arrangements involved two participants of the same sex sharing a room with two beds, which meant that any form of heterosexual intimacy would be difficult to keep private. One well-discussed incident involved rumours of an affair between two volunteers, an angry visit to the residency by the husband of the woman implicated and an altercation between this woman and another woman volunteer around accusations of rumour-mongering. Several weeks later (at T2), we learned that the couple involved had made a complaint to their village elder, and the second woman was fined for ‘assault’. An important point arising from this incident is that the second woman, who was interviewed at T2, felt very unfairly treated and was clear that she would not participate in a residential study again, unless all accommodation was single sex. In addition, this incident was well known within the residence at the time, and many participants noted an associated reputational risk for the KWTRP.

The form that social relations might take in residency seems to have been a concern in some of the communities where volunteers lived. During FGDs, community stakeholders described worries amongst men in their community that their wives might become involved in extramarital affairs during residency. Some clinical staff found it difficult — describing ‘choosing words carefully’ — to explain the study requirement for women volunteers to use contraception during study participation, as this might be interpreted as a tacit acknowledgement and even approval of sexual activities during residency. At the same time, HIS field workers also gave positive accounts of the ways that some families worked together around this potential ‘opportunity’, including one in which a husband and wife both joined the study, at different times, while the other supported the family and wider domestic responsibilities.

From later discussions with community stakeholders, the study had been perceived as contributing to the separation of families in some cases, based on rumours of inappropriate social relations in residency or family disagreements on how to spend compensation payments. mHIS fieldworkers similarly described instances of being confronted by community members whose relationships, including marriages, had deteriorated (including leading to separation) following the participation of one partner in mHIS; in these cases, they described bearing the brunt of the resulting acrimony in families and the wider community. In some cases, an intervention by the KWTRP Community Liaison Group (CLG) was needed to resolve the issue by engaging directly with the community member(s) involved. Reports of these adverse impacts of the compensation payment on families seem to relate to underlying issues of trust or gender inequity:

Also, this issue of money in some households, it had caused conflicts, you’ll find people have had misunderstandings, the husband has...left the in-patient facility and he doesn’t go home, he goes and spend the whole night in a bar, and stays for almost 2 days before reaching home, by the time he gets home he has finished the money, so the conflict now starts between him and his wife. Also, if it’s the wife, she has gotten the money, you see, that is her money now and she says, ‘I’m starting my own business, my own personal project,’ so the husband becomes furious. (Kilifi fieldworker, male, PI3, T3)

#### Wider challenges: community, field workers and research institution

In addition to challenges experienced by volunteers and their families, a range of issues arose with implications for field workers (who are also local residents), the wider community from which mHIS volunteers had been drawn and the research institution and its work.

##### Challenges for fieldworkers

In addition to risks of being blamed when research-related issues emerge for individuals and their families within the community, KWTRP field workers experienced blame for unfairness around study recruitment processes, including accusations of favouring their own families and friends. A fieldworker (and other stakeholders) described being barred from research-related activities in a household where he had earlier assessed a family member as ineligible for the mHIS. Given field workers’ roles as the interface between the research institution and the community, individual issues of trust and blame have clear implications for research institutions.

##### Community concerns

During early community engagement activities, concerns were raised about safety in deliberately infecting volunteers with a disease-causing parasite, related to perceptions of risk and issues around trust in the research process. Specific issues raised at meetings included the severity of illness that might result from deliberate infection, possible future effects on fertility, including infertility or damage to future babies, and concerns that ‘new diseases’ could emerge in the community when volunteers returned home. The relatively high rates of compensation generated by the long residential period fuelled community concerns that the study included high but hidden risks.

As described earlier, the inclusion of HIV testing during screening generated risks of community stigmatisation for volunteers who ‘failed’ to join the study following a health check. This phenomenon illustrates both high levels of awareness across the community about research processes and the stigmatising ways these may be interpreted locally.

…they [community] used to say …you couldn’t join the study, which disease disqualified you? Having in mind HIV/AIDS [giggles]….that’s what disqualified you, and in our community there, HIV/AIDS is not a disease that people are happy about…so whoever will come back, even if its high blood pressure or any other diseases that caused them not to continue with the research, others say it’s HIV (Kilifi community leader, male, IDI5, T3)

##### Institutional harm

KWTRP, as an institution, was implicated by many of the tensions described for families of HIS volunteers, KWTRP field workers and wider communities in Kilifi and Ahero. Negative experiences and rumours around the study carried a risk of undermining trust and generating rumours around the programme’s aims and ways of working. At a practical level, one important challenge concerns the implications of the unusually high levels of compensation given to volunteers in the HIS for recruitment into other community-based studies at KWTRP, and potentially for other research organisations in the area.

First, it brought in challenges to the other studies because of reimbursement…so even now any project or study that is initiated there, someone will ask you, ‘Is that the one where you’ll be admitted at X [guest house used for mHIS]? …if it’s that one then I have no problem but if it’s a different one, ask someone else,’…[or] somebody will tell you, ‘If I participate in this study, will I also be able to participate in the HIS?’ So, if you tell the person that I feel this won’t be possible, then they refuse… (Kilifi field worker, female, PI3, T3)

As described earlier, issues of public trust have been long recognised as a risk for the introduction of HIS in Kenya in general, and Kilifi and Ahero in particular, given the novel and potentially counterintuitive idea that research should involve deliberately giving healthy volunteers with an infection. Earlier papers from KWTRP have described the political and public engagement activities undertaken over many years to assess whether and how these studies should proceed [28]. Similarly, extensive community engagement activities had been undertaken in Kilifi and Ahero prior to the start of HIS in Kilifi in 2017. The issues of individual and community trust in the HIS and KWTRP more widely, has largely been anticipated and community and public engagement strategies developed earlier in the process of planning. One example was the publication of an article in a national newspaper, following completion of phase 1, describing high payments given to volunteers [38]. KWTRP was subsequently inundated with requests from the general public to join the study, prompting a crisis-management response within KWTRP and the KEMRI headquarters in Nairobi, to develop an appropriate response [39].

## Discussion

Drawing on the findings on the nature and extent of benefits and burdens experienced by different research stakeholders in the mHIS, we reflect on the main contributions of our findings to the literature and their implications for policy and practice. While we do not intend to make an assessment of fairness in relation to the balance of benefits and burdens for volunteers in this study, and noting variability across these experiences, we make a set of proposals around ways in which benefits could be further maximised and burdens minimised in the design and implementation of mHIS in low-resource settings.

### The diverse and context-specific nature of benefits and burdens

Taken alongside social science research conducted by our group in Kilifi in T1 and T2, to the best of our knowledge, these studies are the first to explore the nature and extent of benefits and burdens in-depth and across time for HIS stakeholders in a low-resource country. The concept that benefits and burdens experienced by HIS volunteers are likely to go beyond the physical burdens traditionally accounted for during ethics review processes, as shown in this study, has been illustrated in high-resource settings [16–18, 26, 31, 33, 40]. The diverse nature of burdens and benefits has also been shown for different forms of research; a systematic review of qualitative studies around randomised clinical trials (largely in high-resource settings) highlighted the existence of physical, psychological and cost-related burdens, including psychological burdens at every stage in the research process [41].

Our work highlights that the nature of burdens and benefits, across all these domains, is likely to be highly influenced by the wider socioeconomic and cultural context for the HIS, including community perceptions of research staff and the institution. In this low-resource context, while physical burdens were experienced, particularly by those who developed clinical malaria, social, psychological, emotional and economic burdens were amongst the most severe experienced by volunteers and staff, often in interrelated ways. In addition, benefits were perversely linked to burdens, so that enjoyment of the comfortable residential environment and anticipation of using the compensation provided led to sometimes quite marked worries about being forced to leave the residence earlier than hoped.

Our study further highlights that not only are burdens and benefits diverse and context dependent, but they also have important impacts outside the group of volunteers that are traditionally the focus of ethical consideration in research. In our study, families of study participants, the wider communities from which they are drawn, field workers and other staff working in research institutions, as well as research institutions and future studies, may be implicated. While some of the reported individual burdens may appear minor in some situations, repeated and long-term exposure within and across different studies could pose more serious harm to study volunteers, study staff (fieldworkers) and communities involved and increase reputational risks for institutions. These are important areas to consider, for practical and ethical reasons, but are not traditionally well recognised in ethics guidance around research planning.

#### Deliberate infection and trust

The most marked experiences of anxiety for volunteers were generally related to the malaria challenge event, linked to trust in researchers and the research institution, as noted in earlier publications from Kilifi [14, 21]. While volunteers generally understood and supported the social value of the study, they were commonly fearful of the process, as has been described for HIS volunteers in the UK [42, 43]. While anxiety is likely to be at its highest at the time of the challenge injection, long-term follow-up of volunteers shows that these doubts can persist into the long term. Contributing factors were the exact way in which the ‘malaria injection’ was prepared, checked and administered. At this time of extreme anxiety, issues of trust were particularly likely to arise, in contrast to the reliance and appreciation widely expressed for the clinical support provided by the same research clinicians throughout the residency period. These moments of anxiety were fresh in some volunteers’ memories 12–18 months later.

#### The implications of residency requirements

The prolonged period of residency for this mHIS generated many of the most marked social, psychological and economic challenges experienced, as well as determining the high levels of compensation offered. In this largely subsistence economy, volunteers had important — and realistic — concerns over the way that families could be supported financially during residency, particularly where they were the main ‘breadwinner’. Recognition of the economic hardship experienced by some families through giving a lumpsum compensation at the end of residency led to a change in institutional policy; in future studies, such payments will be made on a weekly basis.

More subtly but often to a marked degree, anxiety and sadness arose from worrying about and missing family — particularly children — left at home, and concerns about businesses and other home, family and community commitments left unattended. Some of these worries were well-placed, in that jobs and other economic opportunities were lost, and family arguments and breakups did take place. It would be difficult to understand to what extent research participation contributed to these adverse outcomes, and a modest improvement in economic status was fairly common, if not universal. Nonetheless, efforts should be made to minimise these risks.

Indirectly related to the prolonged period of absence from home, one of the most important social and psychological burdens emerging from this study — impacting volunteers, field workers, communities and the research institution itself — was the nature of relationships that developed during residency. While some relations were clearly individually valued and widely seen as positive, the emergence of intimate relationships involving married men and women in residence generated sometimes serious short- and long-term conflicts. From this study, we cannot know how commonly these relationships developed in residence, exactly which kinds of relationships would be seen as acceptable and not acceptable to other residents, and to what extent these patterns of relations differ from those in life ‘outside’ the residency or are related in some way to the unusual circumstances volunteers find themselves in. On the one hand, we risk being paternalistic in trying to ‘police’ the nature of social relations formed, but on the other, we risk causing offence to other volunteers, family and community conflict and institutional damage by ‘turning a blind eye’. We do know that there were serious repercussions from the incident that we heard most about, for the individuals involved, their families, KWTRP fieldworkers and potentially research activities across the research institution as well as its reputation.

#### The importance of context: social, economic and political influences

The wider socioeconomic and political context within a community ‘hosting’ research and the nature of the existing relationship between the community and the research institution are clear influences on perceptions and experiences of research locally. We have noted that the challenging socioeconomic environment in both counties led to compensation payments being seen as highly desirable, where levels of daily remuneration from economic activities were low, irregular or absent. As earlier mentioned, this background context of economic hardship also made lumpsum compensation payments highly attractive to and challenging for families living on a daily wage. We have also shown that gendered attitudes within families — particularly in coastal Kenya, traditionally a strongly patrilineal culture — could also generate family conflict through disagreement on how funds should be used or who had a right to decide.

Furthermore, serious social, economic and psychological burdens were experienced by the group of volunteers from Ahero related to the unforeseen circumstances linked to the presidential elections when the mHIS was being initiated in that setting. While the study team worked hard to ensure that Ahero participants were supported over this period, particular issues were generated by the need to put recruitment on hold temporarily, to facilitate transport of potential volunteers to Kilifi in a more rushed way than planned (leaving little time to set up systems to manage responsibilities while away) and to conduct a second round of screening in Kilifi to exclude those who had developed malaria in the interval, meaning that some individuals who travelled did not proceed with the study.

#### Willingness to participate in future

When we consider mHIS volunteers’ attitudes over time, it was clear that overall, the majority valued the ‘total package’ offered and were almost universally willing to participate in other studies of this type in future. These positive aspects of participation resonate with those of volunteers in a malaria HIS in the USA; a phase II double-blind, randomised, placebo-controlled trial of a novel antimalarial compound, where volunteers were resident for 2–3 days [24]. Much as for the Kilifi mHIS, volunteers valued the relationships formed with researchers and other participants, appreciated the compensation they received and found challenges around the frequency of blood sampling and the time commitment involved. Arguably, volunteers in Kenya face daily economic challenges of a far more severe nature than those in the USA study, which would be of importance in considering whether willingness to join a study should be seen as a marker of a fair balance between study benefits and burdens. *In a separate paper, we consider the issue of fairness in this balance but here note that many participants described their interest in joining another similar study in future.*

#### Impacts on families, communities and research staff and institutions

Across the findings, it is clear that benefits and burdens to study volunteers in HIS may implicate their families and wider communities as well as research staff and the institution itself, often in interrelated ways. There was often a straightforward direct benefit to volunteers’ families of the cash compensation payments, and less obviously, the knowledge gained by volunteers during residency. But a second, often perverse effect of benefits experienced by volunteers were emerging issues of conflict and trust within families and/or the wider community, as noted across the “Findings” section. In this way, the development of some forms of social relations in residency caused conflict in families, and compensation payments made to women volunteers could undermine cultural traditions, in which male household heads are traditionally family ‘breadwinners’. Similarly, any aspects of the study that generated issues around trust in the research team (the deliberate infection, the frequent blood sampling) could be translated into wider issues of trust in the research institution, its staff and its future research plans. Beyond the more immediate benefits of HIS participation reported in this study, it is important to highlight that in the long term, HIS can contribute to improving individual and community health, in the event of a successful vaccine trial.

### Implications of study findings for the design and implementation of malaria HIS

Throughout this paper, emphasis has been placed on the importance of designing and implementing studies in a manner that maximises the benefits and minimises the risks, burdens and harms for participants, in keeping with international research and HIS-specific ethics guidance [16, 17] and including the need to continuously monitor, assess and document burdens [33]. As highlighted by Rid and Wendler [26], the ultimate goal of the ethical requirement to reduce/minimise risks in clinical research is to reduce harm. Therefore, when harms can be reduced directly through modification of study procedures and requirements in a manner that does not affect the science or undermine the social value of the study, that course of action should be pursued. An important example was the change made by the Kilifi HIS research team to the timing of study compensation payments for future planned research.

This study also shows that the nature of these benefits and burdens can reflect fundamental challenges in designing these studies. Many of the potential burdens of a *requirement* for a residential period are well recognised but ensuring that efforts to compensate for these appropriately is challenging practically and ethically, deeply dependent on the socioeconomic and cultural context and may work in perverse ways. Illustrations provided in this study include deep anxieties around leaving the residency early, the emergence of social relations that later proved to be damaging, and the way that health checks were both valued and feared. There may not be a clear way of balancing burdens and benefits against each other, and careful and in-depth consultation with local stakeholders will be important to finding a good compromise.

It is therefore important to recognise that policies and practices for sensitive research like the Kilifi mHIS need to be developed through careful processes of engagement and a deep understanding of context. In Table 6, we outline a set of considerations and possible ways forward for the issues raised in this discussion, in our context. These issues have been discussed within the main HIS and social science teams together, to plan ways in which the proposed recommendations could be implemented in upcoming HIS at our site. This has been a long-standing collaborative approach between social scientists and clinical scientists within the KWTRP that has seen an increasing number of embedded social science studies within ongoing clinical studies at the programme. Some changes have already been implemented based on our proposed recommendations; for example, the careful development of strategies to support communication on the use of an effective contraceptive by female volunteers before and during the study. Other recommendations are being discussed for longer term implementation, alongside the specific scientific and safety requirements of future studies. Overall, the openness in the responses across our different category of the research participants and the transparency in our descriptions reflect the long-standing respectful relationship our social science research team has built with these different stakeholders. For example, our team members interacted extensively with the mHIS volunteers while in residence, long before the interviews and group discussions were conducted.

**Table 6:** Considering ways of countering burdens and maximising benefits in malaria HIS

Issues	Implications for policy and practice
**Malaria HIS volunteers**	
Physical and psychological burdens during and after the challenge event	• Important to recognise that this is a specific time when volunteers will feel very anxious. Clinical staff may also feel anxious; this is the time they are watching carefully for reactions and have equipment ready for resuscitation if needed. Both groups may need particular support, and staff must be able to communicate clearly and in ways that are trust building with volunteers. • Skilled counselling support for volunteers may be particularly needed at this stage and should be available throughout residency. • Informed consent processes should include more realistic information about this stage, ideally drawing on the account of past volunteers, so that there are no surprises for new volunteers. • There is a need for openness and careful communication around all study procedures, and nothing should be done ‘behind closed doors’. Clinical staff should be aware of the kinds of concerns that volunteers typically have. • Laboratory tours conducted later in residency period created a lot of trust; consider doing this and other similar trust-building activities earlier.
Social, psychological and economic burdens associated with residency	• Skilled counselling support should be available to volunteers throughout residency. • Informed consent processes should include more realistic information about this stage, ideally drawing on the account of past volunteers, so that there are no surprises for current volunteers. This might include video material used as part of engagement with potential volunteers. • Residency should be kept to an absolute minimum period and alternatives considered where possible/meet safety requirements (for example, facilitating more open residency arrangements in local hotels to facilitate follow-up and safety checks; considering the provision of same gender accommodation). • As much movement in and out of a research-run residency as can be managed and is safe (including third party risks) should be allowed, to include volunteers having time ‘out’ and families having time ‘in’. • Compensation payments should be made on a regular basis, not as a lumpsum, at intervals to be determined through community engagement. • Additional support should be given for mobile phone communication with families during residency (not part of compensation). • Consider setting up capacity strengthening activities during residency that would maximise benefits of participation e.g. financial/business management training, and other practical skills.
Preparing for residency and psychological burdens linked to taking contraceptives	• More support needed to individuals and their families in preparing for long residency periods, particularly when volunteers need to travel long distances from home to reach the residency. Particular need to limit repeated health checks or conducting these after travel to the research site, where this is involved. • Clearer communication/engagement and ongoing support around the need for contraception in women volunteers.
**Study staff**	
Preparing study staff, especially field workers to manage challenges arising from recruitment and post-study community dynamics	• Consider developing and undertaking targeted training of fieldworkers on basic problem solving, conflict resolution and effective communication skills. • Consider providing counselling support for study staff, especially fieldworkers, prior to study commencement. • Consider developing a standard response to study staff, especially clinicians responsible for consenting on the need and importance of the study requirement of mandatory enrolment on an effective contraceptive for female volunteers.
**Institutional issues**	
Institutional readiness to respond to potential study-related crisis	• The study team and institution should consider developing a crisis response plan in the event of a crisis emanating about the study in the community or broader public. • Consider targeted engagement with specific sectors of the public, including the media.

## Conclusion

In assessing ways of maximising benefits and minimising burdens in HIS, a grounded account of volunteers, study staff and the wider community members’ experiences provide critical information, understood in the socioeconomic, political and cultural context where studies are implemented. Recognition of the diverse — and sometimes perverse — nature of potential benefits and burdens in a given context, and who this might implicate, is critical to designing studies in ways in which benefits can be maximised and burdens minimised. Community engagement, using approaches that allow well-informed and considered judgements to be made around complex and often unfamiliar proposals for this type of research, is core to research design. For novel and unfamiliar study approaches, like HIS, study design should be informed by prior and embedded forms of social science research that surface the nature and underlying influences for benefits and burdens experienced across populations, over time.

## Supplementary Information

**Additional file 1.** Data collection tools.

## Data Availability

Data that may be made available include data included in the manuscript in form of quotes, summaries of the main themes, and anonymised data transcripts of participant interviews and group discussion, in keeping with the KEMRI-Wellcome Trust Research Programme’s Data Governance Policy. Requests can be facilitated by contacting the KWTRP’s Data Governance Committee on email at dgc@kemri-wellcome.org.

## Abbreviations

HIS: Human Infection Study
mHIS: Malaria human infection study
KEMRI: Kenya Medical Research Institute
KWTRP: KEMRI Wellcome Trust Research Programme
FGD: Focus group discussion
IDI: In-depth interview

## Footnotes

1 KEMRI CCR: https://www.kemri.org/centre-for-clinical-research-ccr-nairobi/

2 See https://kemri-wellcome.org/the-programme

3 Data from T1 and T2 have also been reported in Jao et al., (2020) [14]

4 School Leavers Attachment Scheme: https://sep.kemri-wellcome.org/school-leavers-attachment-scheme/
