# Supplementary material for: Understanding the benefits and burdens associated with a malaria human infection study in Kenya: experiences of study volunteers and other stakeholders
Source: Trials. 2021 Jul 26;22:494. doi: 10.1186/s13063-021-05455-7 (PMC8313115; doi:10.1186/s13063-021-05455-7)
Supplement: Supplementary file 1 — Additional file 1. Data collection tools. [file 13063_2021_5455_MOESM1_ESM.docx]

**ADDITIONAL FILE 1: DATA COLLECTION TOOLS**

**T1&2 STUDY TOOLS**

**FGD Guide for Participants**

1. **FAMILIARIZATION WITH PARTICIPANTS/ICE BREAKER (5 MIN)**
2. Who informed you about this malaria challenge study?
3. What information were you given about this study before starting the screening process?

***(Probe for* what is the study aiming to find out, *screening, consent processes, challenge processes, end of study)***

1. **INFORMED CONSENT PROCESS (10 MIN)**
2. Thinking about all the information giving/screening/consenting sessions you have attended **a)** what are your views about the information given during the information giving sessions? **b)** What was done differently in those different visits? **c)** Was the information given by clinicians and FWs the same?

***(Probe about clarity of the information they were given, how consistent the messaging was, were they able to understand the information? e.g. explanations and demonstrations on blood draw).***

1. Please mention what you know about what the study involves.

***(Do not probe; participants to spontaneously mention screening, consenting, enrollment, in-patient confinement at Pwani Univ., injection with parasites, blood draws and monitoring, ending of study)***

1. What are some of the reasons one may not be able to participate in the study after going through screening? How do you feel about that?

***(Probe for screening failure or qualifying at screening, but not being called to participate).***

1. **DECISION MAKING (80 MIN)**
2. **Motivating factors and barriers (20 min)**
3. What are the reasons that made you want to participate in the malaria challenge study?

***(If not mentioned spontaneously probe on altruism, compensation)***

1. How did the requirements for participating in the study affect your decision making?

(***Probe about*** ***requirements for participation e.g. use of ITNs, for female participants use of contraceptives, 21-day confinement, staying 3 extra days when requiring to leave the study)***

1. How do you feel about being in the study? Any motivating factors? Do you have any regrets? Any change in perceptions between before joining the study and now?

***(Probe for likes, dislikes, concerns or fears)***

1. What importance/value do you think participants place in their involvement in such a challenge study? How does that affect perceptions towards involvement in the study? How does it affect their motivation to participate in the study?
2. **Involvement of family members/significant others (30 min)**
3. Did you disclose your participation in this study to family members/significant others? If not what was the reason?
4. For those who disclosed their participation to family members/significant others, how did you explain the study to them? How did they react?
5. Were there any concerns raised by family members/significant others regarding your participation in the study? What are some of these issues/concerns?

***(Probe for risks, fears, health concerns, absence from home, absence from work and others. Is this experienced differently for female and male participants?)***

1. What reasons/explanations did you give to them as to why you wanted to join the study?
2. Who amongst family members/significant others was involved in or influenced your final decision? Why them? Anyone else?

***(Is this what normally happens when making decisions?)***

1. For those who did not get support from family/significant others for participating in the study, what implications did participating have on your relationships with family, relatives and friends?

***(Is this experienced differently for female and male participants?)***

***break***

1. **Cost of participating in the study (30 min)**
2. Thinking about yourself and others who have participated, what factors do you think had to be weighed/considered when making the decision to participate in this study?

***(Probe about time, procedures, compensation, safety concerns, anything else? Is this experienced differently for female and male participants?)***

1. What factors made it easier for one to make the decision to participate in the study? *Ni* ***(Probe for both study related and family/social factor; is this experienced differently for female and male participants?)***
2. What factors made it difficult to make the decision to participate in the study? ***(Probe for both study related and family/social factors; is this experienced differently for female and male participants?)***
3. What aspects of your life did you think were going to be most affected by your participation in this study and how? ***(Probe about marital, other family/social, work or business related issues)***
4. Being a participant now, what implications do you think this kind of study has on the participants’ lives? ***(Probe for before, during and after the study: Family relations e.g. child care? Other social relations? Work/business?*** ***Any other responsibilities? Is this experienced differently for female and male participants?)***
5. **CONCEPTS OF THE CHALLENGE STUDY (15 MIN)**
6. As you are aware, this study required that participants are injected with the malaria parasite, observed and then treated with anti-malarials once they develop the malaria.
   1. What do you personally think of the idea of infecting people with pathogens in research? ***(probe for pros and cons, and how community might feel/understand this concept?***)
   2. If the research centre is to continue with this kind of research, what might be some of the issues? Why might these arise? How can these issues be addressed?
   3. What might be the risks of directly infecting people with pathogens? What can be done about these risks?
   4. Whose role/responsibility is to ensure that the research is safe for participants? Why do you say so?
   5. On what types of diseases can research of this nature (where people are deliberately infected with the diseases-causing organs) be done? What makes you say so?
   6. Generally, what do you think about the idea of injecting healthy people with malaria parasites and observing the outcome?

***(Assess for perception of risk involved)***

- 1. What are the concerns regarding this type of study from the community?

1. **FUTURE STUDIES (5 MIN)**
2. Would you be willing to participate in a similar study (challenge) in the future?
3. How would you feel about a family member, relative or friend participating in this kind of study? What are your reasons?
4. If anything was to change about how the study is conducted, what would it be?

**FOLLOW UP IN-DEPTH INTERVIEW GUIDE – PARTICIPANTS**

Thank you for allowing me to conduct an/another interview with you today. I am aware that the in-residence stay has been over for a while now, but I would like to talk to a few participants just to follow up on them. I would therefore like to ask you a few questions regarding your experiences during and after the study.

How long did you stay at the in-house facility? What was the experience like? ***(Probe about what s/he liked, what were the challenges faced, how those challenges were resolved/not resolved etc)***

Were there new processes/procedures that were introduced during the study that you were not aware of? What were those? How did you feel about them? What suggestions might you have (Probe: introducing KRA)

Participation in research is voluntary. Reflecting on your participation in the CHMI study so far:

What do you feel about your decision to join the study? (probe, given that you have experienced being in the study, would you make the same decision? If yes or no, why? Do you regret/not regret joining the study?

What do you think about being deliberately being infected with the malaria parasite? Many people were quite apprehensive about it, in reflection, what do you think?

At any time of the study, you could have chosen to withdraw, did you ever consider this option? Why/why not? At what point did you consider? What made you consider that option? Did you share this with anyone else/with clinical team? What made you not withdraw from the study at that time?

What would you say about the voluntariness/right to withdraw for the malaria challenge study? At what point is it possible to withdraw? What were the limitations for withdrawing from the study?

Now that you have been back at home, please tell me how it was for you in terms of settling back home back and returning to your normal routines? ***(Probe any challenges? Whether explained to family and community where they went to? views others have about participating in these types of research;***

What concerns would you say you had or still have? ***(If not spontaneously mentioned tactfully follow up on health status of the participant and whether there are any health concerns);*** Are there any concerns that significant others, other family members or relatives had or still have?

I would like to know what your thoughts are about why you were given the compensation money. What can you say about the levels of compensation? How did you use your compensation money?

1. Did participating in the study have any implications on your life? ***(Ensure to probe for both positive and negative; before, during and after the study i.e. work/business/school;*** ***family relations e.g. with spouse, child care; other social relations; any other responsibilities?)***

Would you say you have any regrets about participating in this study?

Would you be willing to participate in another research of this kind in future?

**FGD GIDE FOR KEMRI COMMUNITY REPRESENTATIVES (KCRS)**

1. **FAMILIARIZATION WITH PARTICIPANTS/ICE BREAKER (5 MIN)**
2. How did you find out about this study, who informed you?
3. What information were you given about this study? ***(Probe for* what the study is aiming to find out, *screening, consent processes, challenge processes, end of study)***

*At this point if need be (based on how part A goes) give brief information about the malaria challenge study (5 min). Then to continue with Part B.

1. **INFORMED CONSENT PROCESS (15 MIN)**
2. Have you attended any community information giving sessions for this study yourself? What would you say worked well?

***(Probe about clarity of information they were giving, how consistent the messaging was, were community members understanding the information? e.g. explanations and demonstrations on blood draw, screening failure)***

1. Thinking about the information given in the community, are there areas regarding the study that community members seem to have a challenge in understanding or accepting?
2. What do you think are the issues that have been most challenging for study staff to discuss or respond to? How have these been dealt with?
3. **DECISION MAKING (80 MIN)**
4. ***Motivating factors and barriers (25 min)***
5. What are some of the factors that made people decide to participate or not to participate in the malaria challenge study?

***(Probe about time, procedures, compensation, safety concerns, anything else? Is this experienced differently for female and male participants?)***

1. How do you think the requirements for participating in the study affected participants’ involvement in the study?

***(Probe about requirements e.g. use of ITNs, for female participants use of contraceptives, compensation levels, 21-day stay at the hostel away from home, staying 3 extra days when requiring to leave the study)***

1. What have been the main concerns raised by the participants regarding the study about the screening/consenting sessions? During the study? After the study?

***(Probe for expected and unexpected)***

1. What importance/value do you think participants place in their involvement in such a challenge study? How does it affect how you handle your roles in the study? How does it affect their motivation to participate in the study?
2. **Involvement of family members/significant others (20 min)**
3. Do you know anyone who participated in a malaria challenge study? Would you know whether participants disclose their participation in the challenge studies to family members/significant others? Why?
4. Has anyone ever consulted you about participating in a malaria challenge study? Who was it? ***(Probe about relationship, gender, age, occupation)*** What reasons/explanations were given to you and significant others as to why s/he wanted to join the study? ***(especially for female participants)?*** How did you react?
5. What concerns might family members/significant others raise regarding participants’ involvement in the malaria challenge study?

***(Probe for risks, fears, health concerns, absence from home, absence work and others. Is this experienced differently for female and male participants?)***

1. How might this affect participants’ decision making for participation? Is this what normally happens when making decisions for involvement in research in this community?
2. For those who did not get support for involvement in the study, what implications might their participation have on their relationships with family, relatives and friends?

***(Probe is this experienced differently for female and male participants?)***

1. ***Cost of participating in the study (30 min)***
2. What do you think people have to weigh/balance as they made the decision whether or not to participate in this malaria challenge study?

***(Probe for study related and family/social factors; is this experienced differently for female and male participants?)***

1. What factors make it easier for one to make the decision to participate in a malaria challenge study?

***(Probe for both study related and family/social factor; is this experienced differently for female and male participants?)***

1. What factors make it difficult to make the decision to participate in a malaria challenge study?

***(Probe for both study related and family/social factors; is this experienced differently for female and male participants?)***

1. What implications do you think involvement in the study has on the participants’ lives? How were these solved/how did they cope with this?

***(Probe for before, during and after the study: Family relations e.g. child care? Other social relations? Work/business?*** ***Any other responsibilities? Is this experienced differently for female and male participants?)***

1. **CONCEPTS OF THE CHALLENGE STUDY (15 MIN)**
2. As you are aware, this study required that participants are injected with the malaria parasite, observed and then treated with anti-malarials once they develop the malaria.
3. What do you personally think of the idea of infecting people with pathogens in research?

***(probe for pros and cons, and how community might feel/understand this concept?***)

1. If the research centre is to continue with this kind of research, what might be some of the issues? Why might these arise? How can these issues be addressed?
2. What might be the risks of directly infecting people with pathogens? What can be done about these risks?
3. Whose role/responsibility is to ensure that the research is safe for participants? Why do you say so?
4. On what types of diseases can research of this nature (where people are deliberately infected with the diseases-causing organs) be done? What makes you say so?
5. Generally, what do you think about the idea of injecting healthy people with malaria parasites and observing the outcome?

***(Assess for perception of risk involved)***

1. What are the concerns regarding this type of study? From staff/community?
2. **FUTURE STUDIES (5 MIN)**
3. Would you be willing to participate in a similar study (challenge) in the future? What do you think about a family member, relative or friend participating in this kind of study? What are your reasons?
4. Do you think community members will be willing to participate in a similar (challenge) study in the future?
5. If anything was to change about how the study is conducted, what would it be?

**FGD Guide for Field Workers**

1. **ICE BREAKER (5 MIN)**
2. Briefly explain the aim of the malaria challenge study?
3. What are your roles in the Malaria challenge study?
4. What information were you giving community members about this study before inviting them for the screening process?

***(Do not probe, look out for spontaneous mention of:* what the study is aiming to find out, *screening, consent processes, challenge processes, end of study)***

1. **INFORMED CONSENT PROCESS (15 MIN)**
2. Thinking about the information giving sessions in the community, **a)** what worked well? **b)** what are the issues that have been most challenging to discuss or respond to? How have these been dealt with?

***(Probe: Are there areas regarding the study that community members seem to have a challenge in understanding or accepting?)***

1. What have been your experiences of giving information comparing **a)** between the first and subsequent information giving sessions in this study? ***(Probe about clarity of information they were giving, how consistent the messaging was, were community members understanding the information? e.g. explanations and demonstrations on blood draw, screening failure)* b)** comparing this study and other types of studies you have been involved in?
2. Comparing this study and others studies, what can you say about **a)** consent levels (high, low, about the same) **b)** retention levels **c)** understanding levels; and why these levels?
3. **DECISION MAKING** **(80 MIN)**
4. **Motivating factors and barriers (25 min)**
5. What are some of the factors that made people decide to participate or not to participate in the malaria challenge study?

***(Probe about time, procedures, compensation, safety concerns, anything else? *Is this experienced differently for female and male participants?)***

1. How do you think the requirements for participating in the study affected participants’ involvement in the study?

***(Probe about requirements e.g. use of ITNs, for female participants use of contraceptives, compensation levels, 21-day stay at the hostel away from home, staying 3 extra days when requiring to leave the study)***

1. What have been the main concerns raised by the participants regarding the study about the screening/consenting sessions? During the study? After the study?

***(Probe for expected and unexpected)***

1. What importance/value do you think participants place in their involvement in such a challenge study? How does it affect how you handle your roles in the study? How does it affect their motivation to participate in the study?
2. **Involvement of family members/significant others (25 min)**
3. Would you know whether participants disclosed their involvement in the malaria challenge study to family members/significant others? Why?
4. What concerns might family members/significant others raise regarding participants’ involvement in the malaria challenge study?

***(Probe for risks, fears, health concerns, absence from home, absence from work and others. Is this experienced differently for female and male participants?)***

1. How might this affect participants’ decision making for participation? Is this what normally happens when making decisions for involvement in research in this community?
2. For those who did not get support for involvement in the study, what implications might their participation have on their relationships with family, relatives and friends?

***(Probe is this experienced differently for female and male participants?)***

1. **Cost of participating in the study (30 min)**
2. What do you think people had to weigh/balance as they made the decision whether or not to participate in this malaria challenge study?

***(Probe for study related and family/social factors; is this experienced differently for female and male participants?)***

1. What factors make it easier for one to make the decision to participate in a malaria challenge study?

***(Probe for both study related and family/social factor; is this experienced differently for female and male participants?)***

1. What factors make it difficult to make the decision to participate in a malaria challenge study? ***(Probe for both study related and family/social factors; is this experienced differently for female and male participants?)***
2. What implications do you think involvement in the study has on the participants’ lives? How were challenges solved/how did they cope with this?

***(Probe for before, during and after the study: Family relations e.g. child care? Other social relations? Work/business?*** ***Any other responsibilities? Is this experienced differently for female and male participants?)***

1. **CONCEPTS OF THE CHALLENGE STUDY (15 MIN)**
2. As you are aware, this study required that participants are injected with the malaria parasite, observed and then treated with anti-malarials once they develop the malaria.
3. What do you personally think of the idea of infecting people with pathogens in research?

***(probe for pros and cons, and how community might feel/understand this concept?***)

1. If the research centre is to continue with this kind of research, what might be some of the issues? Why might these arise? How can these issues be addressed?
2. What might be the risks of directly infecting people with pathogens? What can be done about these risks?
3. Whose role/responsibility is to ensure that the research is safe for participants? Why do you say so?
4. On what types of diseases can research of this nature (where people are deliberately infected with the diseases-causing organs) be done? What makes you say so?
5. Generally, what do you think about the idea of injecting healthy people with malaria parasites and observing the outcome?

***(Assess for perception of risk involved)***

1. What are the concerns regarding this type of study? From staff/community?
2. **FUTURE STUDIES (5 MIN)**
3. Do you think community members will be willing to participate in a similar (challenge) study in the future?
4. Thinking about the information giving sessions, what more information could have been given to participants

***(for CE, by field workers before screening, during screening and during study?)***

1. If anything was to change about how the study is conducted, what would it be?

**IN-DEPTH INTERVIEW GUIDE – PRINCIPAL INVESTIGATOR (2)**

1. **ICE BREAKER**

Thank you for allowing me to conduct another interview with you. I am aware that the in-residence stay for cohort 3 challenge has been over for a while now. So what is happening with the study right now?

1. **GENERAL EXPERIENCES/LEADERSHIP**

What are your perceptions of how the study performed ***(Probe whether successful or not successful, what made the study to succeed or not; what this means?***

1. What has been the most exciting experience in leading this study during cohort 3 challenge?

What was challenging in the conduct of this study? How does this differ with previous experience? What did you do about the challenges? ***(If not spontaneously mentioned probe about differences emanating from involving Kilifi and Ahero participants).***

Are there any particular issues that were unique to participation of this study? Why did these arise? ***(Probe about issues related to the work of frontline staff, technical staff, participants, PI herself, involving the 2 study sites)***

1. **INFORMED CONSENT PROCESS**

(If not mentioned above) Some FGD participants had the perception that the selection criteria and recruitment processes were not clear and fair. What would you say about that?

1. This is a unique study model with the potential of various aspects about it creating motivation for participation; including the compensation levels. You are aware that already there has been increased ‘appetite’ for this study in the community. Would you say this is affecting the autonomy of the participants?
2. **DECISION MAKING**
3. There was a general perception that some of the inclusion criteria requirements had implications on decision making for participating in the study, one of them being the requirement for female participants to be on contraceptives. What are your thoughts about that?

There are issues that raised during the qualitative study that indicate there was a delicate balance between motivation for participating in the study and issues of trust towards the institution. What are your thoughts about that?

1. **COST OF PARTICIPATION**

Some participants expressed dissatisfaction with some study procedures especially when information provided had not been earlier understood or when new information came up during the course of the study. What do you think about their dissatisfaction with:

Not having any upkeep money during the study i.e. not being able to afford credit to call home, basic grooming e.g. visiting the barber shop etc.

Perceived unfair treatment at the in-house facility e.g. irregular access to mineral water, poor treatment of CHMI participants whenever other visitors were at the guest house?

Lack of clarity with the length of stay at the in-house facility?

Lack of clarity about how the days for compensation would be calculated?

KRA registration?

**FUTURE STUDIES**

1. What have you learnt from this phase of the study that is becomes an opportunity for making adjustments in the implementation of future studies? ***(Probe for issues related to information giving, recruitment, handling of participants during their in-house stay)***

What do you think about suggestions that have been made by FGD participants concerning:?

Providing financial management training to participants for better handling of their compensation money?

- Offering counselling services to reduce conflicts amongst participants?
- Separating male and female participants to reduce temptations for developing sexual relationships amongst them?

**IN DEPTH INTERVIEWS (IDI) GUIDE – CLINICIANS**

1. **ICE BREAKER (5 MIN)**
2. Briefly explain the aim of the challenge study.
3. What are your roles in the Malaria challenge study?
4. **INFORMED CONSENT PROCESS (10 MIN)**
5. Thinking about the information giving, screening and consenting sessions what **a)** worked well? **b)** what are the issues that have been most challenging to discuss or respond to? How have these been dealt with? ***(Probe: Are there areas regarding the study that community members seem to have a challenge in understanding or accepting?)***
6. What have been your experiences of giving information comparing **a)** between the first and subsequent information giving sessions in this study? ***(Probe about clarity of information they were giving, how consistent the messaging was, were community members understanding the information? e.g. explanations and demonstrations on blood draw, screening failure)* b)** comparing this study and other types of studies you have been involved in?
7. Comparing this study and others studies, what can you say about **a)** consent levels (high, low, about the same) **b)** retention levels **c)** understanding levels; and why these levels?
8. **DECISION MAKING (30 MIN)**

**Motivating factors and barriers (20 min)**

1. What are some of the factors that make people decide to participate or not to participate in the malaria challenge study? ***(Probe about time, procedures, compensation, safety concerns, anything else? *Is this experienced differently for female and male participants?)***
2. How do you think the requirements for participating in the study affected participants’ involvement in the study? Probe about ***requirements e.g. use of ITNs, for female participants use of contraceptives, staying 3 extra days when requiring to leave the study)***
3. What have been the main concerns raised by the participants regarding the study during the screening/consenting sessions? During the study? After the study? ***(Probe for expected and unexpected)***
4. What importance do you think participants place in their involvement in such a challenge study? How does it affect how you handle your roles in the study? How does it affect their motivation to participate in the study?
5. **Involvement of family members/significant others (20 min)**
6. Would you know whether participants disclosed their involvement in the malaria challenge study to family members/significant others? Why?
7. What concerns might family members/significant others raise regarding participants’ involvement in the malaria challenge study? ***(Probe for risks, fears, health concerns, absence from home, absence work and others. Is this experienced differently for female and male participants?)***
8. How might this affect participants’ decision making for participation? Is this what normally happens when making decisions for involvement in research in this community?
9. For those who did not get support for involvement in the study, what implications did participating have on their relationships with family, relatives and friends? ***(Is this experienced differently for female and male participants?)***
10. **Cost of participating in the study (20 min)**
11. What do you think people had to weigh/balance as they made the decision whether or not to participate in this malaria challenge study? ***(Probe for study related and family/social factors; is this experienced differently for female and male participants?)***
12. What factors make it easier for one to make the decision to participate in a malaria challenge study? ***(Probe for both study related and family/social factor; is this experienced differently for female and male participants?)***
13. What factors make it difficult to make the decision to participate in a malaria challenge study? ***(Probe for both study related and family/social factors; is this experienced differently for female and male participants?)***
14. What implications do you think involvement in the study has on the participants’ lives? How were challenges solved/how did they cope with this? ***(Probe for before, during and after the study: Family relations e.g. child care? Other social relations? Work/business?*** ***Any other responsibilities? Is this experienced differently for female and male participants?)***
15. **CONCEPTS OF THE CHALLENGE STUDY (10 MIN)**
16. This study required that participants are injected with the malaria parasite, observed and then treated with anti-malarials once they develop the malaria.
17. What do you personally think of the idea of infecting people with pathogens in research? ***(probe for pros and cons, and how community might feel/understand this concept?***)
18. If the research centre is to continue with this kind of research, what might be some of the issues? Why might these arise? How can these issues be addressed?
19. What might be the risks of directly infecting people with pathogens? What can be done about these risks?
20. Whose role/responsibility is to ensure that the research is safe for participants? Why do you say so?
21. On what types of diseases can research of this nature (where people are deliberately infected with the diseases-causing organs) be done? What makes you say so?
22. Generally, what do you think about the idea of injecting healthy people with malaria parasites and observing the outcome? ***(Assess for perception of risk involved)***
23. What are the concerns regarding this type of study? From staff/community?
24. **FUTURE STUDIES (5 MIN)**
25. Do you think community members will be willing to participate in a similar (challenge) study in the future?
26. Thinking about the information giving sessions, what more information could have been given to participants ***(for CE, by field workers before screening, during screening and during the study?)***
27. If anything was to change about how the study is conducted, what would it be?

**T3 STUDY TOOLS**

**Interview Guide for CE team**

**Focus area**: Community engagement

1. **Easing**

Can you talk to me how community engagement was undertaken during the CHMI study?

1. **Community Engagement Plan**
2. **Planning for community engagement**
3. Did you have a CE plan for the study? If Yes. Will you like to talk to me more about what the plan entailed? (Probe: Who was involved in developing the plan, what role did you play? etc.)
4. Which communities were identified for engaging with? What were the reasons for targeting those communities (how)?
5. How was engagement with different stakeholders undertaken and how different were the approaches used and the response received?
6. Are there communities that you felt should have been considered but were not included in the plans?
7. How/who was to fund the engagement activity?
8. When were the plans for CE to be implemented, and why was that time agreed?
9. Was the CE plan for the CHMI study different from those for other health research such as clinical trials? If Yes, how?
10. **Implementation of the community engagement**
11. Who were those involved in the implementation and why? (*Probe about study investigators, study coordinator, CE staff, CHVs, Clinical staff etc*.). What role was each of these team members required to play?
12. How was the team responsible for implementing the CE plan prepared/capacitated for the tasks they had at hand? (Probe if: any specific training was provided for the planned activities, if specific staff with specialized skills were recruited etc.)
13. **Engagement activities/approaches**
14. What engagement activities/approaches were used and why? With which communities? How did the engagement unfold over time (Probe: were activities implemented as planned? If there were changes that happened, which were those and why?
15. What was the key information/message for the different communities that were engaged? What materials were used for the CE activities?
16. According to you, how effective was the engagement approach in passing the key messages? What makes you say that?
17. **Feedback from CE activities, Challenges and Lessons Learned**
18. **Feedback from implementation**
19. How did the various targeted individuals and groups respond to the CE activities? Were the people interested in the activities? (Probes: Turn-out/ attendance for group events (average no. of people attending group events); Common questions asked during CE activities; Major concerns that were raised and how the team responded; and Plans to provide feedback at the end of study and how etc.)
20. **Challenges and lessons learned from CE**
21. Can you share with me the major challenges that were experienced by the team during the process of CE? (Probes: staff training needs; poor cooperation from community actors; unfavorable pollical environment; limited finance for some activities etc.). How did the team navigate through these challenges?
22. In retrospect, are there some adjustments you would have done to the CHMI-SIKA study CE plan? (Probes: target groups and individuals; key messages; timing of activities etc.) If Yes, what will some of these adjustments be?
23. What are the key lessons that the team has learned from implementing the CE plan for the CHMI-SIKA study? How could these lessons be integrated/incorporated for CE activities for future HIS within the programme?
24. Are there any plans for providing feedback to the study communities post-study?

**Interview Guide for Chief, KCRs & other community leaders**

**Focus areas**

1. Community engagement
2. Community perceptions and dynamics: prior, during and post-study
3. **Community engagement**
4. How was CE for the challenge study undertaken within your community?
5. What role did you play in CE for the challenge study?
6. What engagement activities/approaches were used and why?
7. What were the key information/message that the study team presented during the barazas in the community? What materials were used for the CE activities?
8. According to you, how effective was the engagement approach in passing the key messages? What makes you say that?
9. How did community members respond to the CE activities? Were the people interested in the activities? (Probes: Turn-out/ attendance for group events (average no. of people attending group events); Common questions asked during CE activities; Major concerns that were raised etc.)
10. **Community perceptions and dynamics: prior, during and post-study**
11. As community members, what were the prevailing discussions within the community when the former study participants were away at Pwani University in Kilifi for the challenge study? What did people make of their long stay away from the community? Were there any issues/concerns raised about their long stay? Are you aware of any issues raised by their families?
12. Following the return of the former study participants from Kilifi back to the community, what were the prevailing discussions going on within the community about them? What did people say or feel about them? What attitude did they display about the study within the community? How were they treated by other people within the community?
13. Are you aware of any unintended consequences of the challenge study within the community? Are you aware of any negative things that happened/ are happening in the community because of the challenge study taking place in this community?
14. How do people in the community react or behave when someone is screened but disqualified from joining the study? How do they interpret that?
15. Are you aware how former study participants spend the out-of-pocket compensation they received from the study? What do people in the community make of the compensation that participants receive from the study? What are the community perceptions about the amount that is provided as compensation for the challenge study? Are you aware of any unintended consequences of the compensation on familial and community relations? Are you aware of former study participants who have put their study compensation into very productive use that has drawn positive feedback from the community?

**Interview Guide for Study Investigators**

**Focus areas**

1. Community engagement
2. Recruitment, Screening, Enrolment and Consent processes
3. In-patient stay
4. CHMI implementation: ethics and regulatory issues, barriers, facilitators, challenges, and lessons learned
5. **Community engagement**
6. How was CE undertaken in your area?
7. How was CE for CHMI-SIKA different from that of other clinical research?
8. How was engagement with different stakeholders undertaken and how different were the approaches used and the response received?
9. What were the challenges and facilitators experienced during CE
10. Were there any changes to the initial CE plan and why?
11. What were the lessons learned from CHMI CE?
12. Are they any plans for providing feedback to communities post-study?
13. **Recruitment, Screening, Enrolment and Consent processes**

**Recruitment**

1. What happens before a potential participant makes his/her way for screening (activities and actors within the community)? What was the recruitment strategy?
2. What strategies did the team have in place to ensure **a fair participant recruitment** process? Were they situations where some potential participants/community members raised concerns about the recruitment strategy? How were such incidents addressed?

**Screening**

1. According to the protocol potential participants with abnormal/significant results had to be referred to appropriate medical centres arranged with the permission of the volunteer. In practice, how was this done for the CHMI study? How common was referral for medical attention? How did potential participants react when informed on the need for further referral?
2. What were common questions asked by potential participants prior and during the screening process?
3. Where was the screening undertaken? How was disclosure of major health problems undertaken during the study? Were they any surprises during the screening process that the team had not anticipated? Were there situations where volunteers turned down the medical referral? Were they any issues that the team struggled to manage?
4. Were there situations where potential participants concealed or withheld some information/ lied because of fear of being screened-out? If yes, how common were such situations and how did the study team address them?
5. What were potential participants’ perception of the screening exercise? (objective, reliable, unreliable, could be manipulated etc.). What strategies did the study team have in place to ensure that potential participants trusted the screening exercise and accepted the lab tests?
6. How and when were the screening test results provided to potential participants, and who had the responsibility to provide the test results? How did potential participants typically react when they are informed that they are: (a) eligible and (b) not eligible to enroll for study enrolment?
7. What were the prevailing attitudes of community members towards potential participants who were screened and determined to be not eligible to enrol for the study?

**Enrolment (if applicable)**

1. What happened when participants arrived at the CGMR-C in Kilifi for enrolment? What activities had to be undertaken before they were to be enrolled into the study and who were those responsible for those tasks?
2. What were the challenges experienced by the team during enrolment? Were there any issues that arose during enrolment that the team didn’t anticipate? Any surprises? If yes, how were they addressed?
3. What systems were in place to ensure that all female participants enrolled on contraceptives before study enrolment and continued enrolment throughout the study?

**Consenting**

1. What strategies were in place to ensure that potential participants gave appropriate informed consent (esp. understanding of the risks and discomforts associated with the study) before enrolling on the study? Were there situations where some eligible potential participants did not consent for enrolling in the study following the consenting process? If yes, what were some of the potential reasons provided?
2. Were there situations were enrolled participants wanted to withdraw from the study before study completion? If yes, what were the reasons provided? In such situations, did they stay for the 3 days allocated for clearing the parasites before leaving the in-patient facility?
3. Overall, what were the challenges experienced by the team experience during the consenting process?
4. What lessons were learned by the team with regards to consenting participants for studies like CHMI?
5. **In-patient stay (if applicable)**
6. Tell me about your experience of study participants dynamics during in-patient stay? How did they get along with (a) fellow participants and (b) study team during their stay? How did they resolve disagreements and conflicts while at the in-patient facility?
7. What were their perceptions of and reactions towards (a) the study procedures, (b) long-term in-patient stay with limited mobility? What were some of the concerns raised by participants during their in-patient stay on these issues?
8. What were the burdens/discomforts mentioned by the participants? What do you think were their worst experiences? What about their best experiences (if any)? How did the study team navigate through these issues?
9. As a member of the study team, are they some things that should be considered in future challenge studies to reduce the discomforts/burdens experienced by participants during their in-patient stay?
10. What were the lasting memories that stuck with you while interacting with the participants during their in-patient stay at Pwani University?
11. **CHMI implementation**
12. What are research team’s experiences with research ethics committee(s) and regulatory authorities responsible for reviewing and approving challenge studies in Kenya? (probe how the interactions and responses have been over time; whether there is need to have informal discussions with some members of the RECs/RAs before submitting the protocol; How they have found RECs/RAs understanding and appreciation of challenge studies etc).
13. As CHMI investigators, what lessons that have been learned over the years to support the effective conduct of challenge studies in in LMIC settings like Kenya?

**Interview Guides for Fieldworkers/Community Health Volunteers**

**Focus areas**

1. Community engagement
2. Recruitment and Screening
3. Community perceptions and dynamics: prior, during and post-study
4. **Community engagement**
5. What role did you play in CE for the CHMI-SIKA study (if any)?
6. How was CE for CHMI-SIKA different from that of other clinical studies?
7. How was engagement with Chiefs and communities undertaken and how different were the approaches used and the response received?
8. What were the challenges and facilitators experienced during CE
9. What were the lessons learned from CHMI CE?
10. **Recruitment and Screening**

**Recruitment**

1. What happens before a potential participant makes his/her way for screening (activities and actors within the community)? What was the recruitment strategy?
2. What strategies did the team have in place to ensure **a fair participant recruitment** process?
3. Were there situations where some potential participants/community members raised concerns about the recruitment strategy? How were such incidents addressed?

**Screening**

1. As a community health volunteer (CHV)/fieldworker (FW), what were potential participants’ perception of the screening exercise? (objective, reliable, unreliable, could be manipulated etc.).
2. What were the prevailing attitudes of community members towards potential participants who were screened and determined to be not eligible to enrol for the study?

**Community perceptions and dynamics: prior, during and post-study**

1. As a CHV/FW, what were the prevailing discussions within the community when the former study participants were away at Pwani University-Kilifi for the challenge study?

What did people make of their long stay away from the community? Were there any issues/concerns raised about their long stay? Are you aware of any issues raised by their families?

1. Following the return of the former study participants from Pwani University back to the community, what were the prevailing discussions going on within the community about them? What did people say or feel about them? What attitude did they display about the study within the community? How were they treated by other people within the community?
2. Are you aware of any unintended consequences of the CHMI study within the community? Are you aware of any negative things that happened/ are happening in the community because of the CHMI study taking place in this community? Are you aware of any negative thing that is happening to other studies taking place in this community because of the CHMI study is taking place in the community?
3. How do people in the community react or behave when someone is screened but disqualified from joining the study? How do they interpret that?
4. Are you aware how former study participants spend the out-of-pocket compensation they received from the study?
5. What do people in the community make of the compensation that participants receive from the study?
6. What are the community perceptions about the amount that is provided as compensation for the CHMI study?
7. Are you aware of any unintended consequences of the compensation on familial and community relations?
8. Are you aware of former study participants who have put their study compensation into very productive use that has drawn positive feedback from the community?

**Interview Guide for former CHMI-SIKA study participants**

This interview guide presents questions for former CHMI-SIKA study participants, focusing on the period from *final health check before challenge/consent process/administration of the challenge/follow up period of residence at Pwani University guest house and after the study.* The guide is intended to explore participants understanding/ knowledge about the study and experiences of participation.

***Thank you for allowing us to talk with you today. We are talking to former participants of the KEMRI malaria challenge study to hear their experiences of participating in the study. I would therefore like to ask you a few questions regarding your experience* focusing on the period from *final health check before challenge/consent process/administration of the challenge/follow up period of residence at Pwani University guest house and after the study.***

1. **Introduction/general responses:** It is now many months since the study ended and you returned to the community. How has life been since returning to the community?

*Probe generally including about health, wellbeing etc. Listen for any issue raised spontaneously that are relevant to HIS e.g. about health being better/worse, about SE situation being better/worse. If these are mentioned, acknowledge issue and indicate that you would like to come back to this.*

1. **Participants Understanding/Knowledge about the study:** Can you tell me what you think the main aim of this project/study was? What the reason for the project being set up and run?

*Probe perceptions as research (e.g. learning about a new malaria vaccine, learning about malaria etc in ways that show this learning was for all people, not for participants specifically), and what aim of research was. Note this is current perceptions – where possible compare with perceptions at the time (from previous data) and note any differences*

1. **Participants recall/perceptions of activities at KEMRI during research (final health check/challenge/ residence): what happened and how they felt about this**

Can you tell me what exactly happened **when you came to Kilifi to join this study**, and how you felt about that?

*Allow participant to narrate his/her experiences during the study as below, focusing on the period [before challenge, administration of the challenge and after challenge/follow up period of residence at Pwani guest house - PGH]. Note which activities they remember* ***spontaneously*** *(what order?), and* ***probe*** ***for each area*** *as shown below or in table. After they have talked about all the activities they remember,* ***remind*** *them of any they have not mentioned, and again probe as shown below and in table.*

- 1. **Immediately before challenge & moving into PGH**

**Listen for accounts of activities including:**

- *Final health check*
- *Informed consent process*
- *Anything else?*

**For any procedures they mention spontaneously, ask:**

- *What exactly happened?*
- *How did that make you feel* ***at the time?***
- *[If relevant] What did you do? What was the effect of that (if they took an action)?*
- *Were you told anything about this activity beforehand – what was that?*
- *And how do you feel about this* ***now*** *– has there been any change since then till now? (probe for any changes in how these activities are viewed)*

*Remind participants about any areas not mentioned specifically, and ask questions above about these.*

- 1. **At the time of being given the malaria injection (*being given the challenge injection with close clinical monitoring)***
  - *What exactly happened?*
  - *How did that make you feel* ***at the time****?*
  - *What did you do (if relevant)? What was the effect of that (if they took an action)?*
  - *Were you told anything about this activity beforehand – what was that?*
  - *And how do you feel about that* ***now*** *– has there been any change? (probe for any changes in how these activities are viewed)*
  1. **About the time you stayed in Pwani Guest house** *(including procedures, symptoms, being looked after generally, getting on with staff and other participants)*
- *What are the things you remember most strongly? (explore these as in table below)*
- *What else do you remember? (listen to responses and probe as in table)*
- *Do you remember….? (remind them about other areas and probe again as in table)*

| **About participant’s experiences – areas of activity** | **Probes** |
| --- | --- |
| Procedures/symptoms/treatment after challenge, in residence:   - Blood tests - Medical check-ups - Anything else? | - What exactly happened? - How did that make you feel? (**likes & dislikes**, where latter includes **anxieties, concerns & discomforts**) - What was it particularly about that that you liked/disliked? - What did you do (if relevant)? - What was the effect of that (if they took an action)? |
| Any symptoms & treatment given   - Experience of illness/symptoms - Being given treatment - Anything else? | - What exactly happened? - Can you tell me how that made you feel? *(probe for* ***physical*** *and* ***psychological*** *symptoms e.g. pain and worry)* - How did that compare to illnesses you’ve had in the past? - How long did you feel ill for? *(probe for how long he/she felt very unwell vs recovering)* - Do you have any comments on how it felt taking treatment? (*any problems associated with treatment?)* - How did you feel after taking treatment? *(probe for how quickly improved)* |
| Relationship with other participants | - How did the group of participants generally get on with each other? - Have you kept in touch with anyone there since you left – can you tell me more about that? (Probe – any new friendships?) |
| Relationship with KEMRI staff | - How did you get on with KEMRI staff you met while you were staying at PGH? *(clarify which staff talking about)* - Can you tell me more about that/how did that make you feel? (Probe for **positive** and **negative** experiences) - What were the reasons you felt like this? (probe for personal attitudes/availability/prior relationships etc) |
| Inpatient facility – comfort,  convenience etc | - What did you think about the facilities at PGH where you stayed? (probe for positive and negative accounts) - (where relevant) What could KEMRI have done to make this better? |

1. **Experiences at home following participation: Early and later**

Can you tell me what happened when you came home immediately after leaving PGH? Where did you go and what did you do? (e.g. it must have been great to get back to your normal life?)

What were the things you **most appreciated/enjoyed** about getting home after this time away?

- *Reception by significant others and community after the study?*
- *Any use of compensation?*

Were there any **difficulties** you met when you got home after this time away?

- *Impact on his/her obligations/roles (family, social and work)?*
- *Impact on family and significant others?*

**And now you’ve been home for some tim**e, how has this affected *[refer back to positive and difficult areas mentioned above*]? *Probe to understand how these have worked out over time.*

1. **[If not already discussed] Financial compensation**

Can I ask you about how useful the cash that was given to you as compensation for time was in the end?

- How it was spent
- Effect on family (have you been able to talk to family about it)
- Reason why they think it was given
- Whether they think it was reasonable – including whether and how thinking about this has changed over time

1. **Recommendations**: As a final question, can we talk about areas you’ve mentioned where you met some challenges (note from earlier part of interview) – do you have any recommendations about ways in which you think KEMRI should make changes? Is there a reason you think they should do this? (*specifically – not something else*)

***We have talked about your experiences of participating in the study, do you have any comment or question about what we have talked about or any additional experience about your participation in the study which you would wish to share with us?***

Thank you for taking off time to talk about your experiences of participating in the malaria challenge study. We are also interested to get the views of significant others of former study participants **who were aware of their significant other’s participation** in the challenge study. These may include family and friends. Was any of your significant other aware of your participation in the challenge study? If yes, will it be okay if we approach him/her and request for a short interview of about 30 – 40 minutes? We will mainly explore their perception of this type of research and their experiences while you were away to participate in the study.

If agree, request for the name and contact number of 1-2 significant others. If agreement is not secured, do not request for the contact details. Thank the participant for her time and end the interview.

-END-
